# Chemo-Enzymatic Synthesis of Renewable Sterically-Hindered Phenolic Antioxidants with Tunable Polarity from Lignocellulose and Vegetal Oil Components

**DOI:** 10.3390/ijms19113358

**Published:** 2018-10-26

**Authors:** Louis Hollande, Sandra Domenek, Florent Allais

**Affiliations:** 1 Chaire ABI, AgroParisTech, CEBB 3 rue des Rouges Terres 51110 Pomacle, France; louis.hollande@agroparistech.fr; 2 UMR GENIAL, AgroParisTech, INRA, Université Paris-Saclay, Avenue des Olympiades, 91300 Massy, France; sandra.domenek@agroparistech.fr

**Keywords:** ferulic acid, fatty acid ethyl esters, CAL-B, antioxidant, DPPH

## Abstract

Despite their great antioxidant activities, the use of natural phenols as antioxidant additives for polyolefins is limited owing to their weak thermal stability and hydrophilic character. Herein, we report a sustainable chemo-enzymatic synthesis of renewable lipophilic antioxidants specifically designed to overcome these restrictions using naturally occurring ferulic acid (found in lignocellulose) and vegetal oils (i.e., lauric, palmitic, stearic acids, and glycerol) as starting materials. A predictive Hansen and Hildebrand parameters-based approach was used to tailor the polarity of newly designed structures. A specific affinity of *Candida antarctica* lipase B (CAL-B) towards glycerol was demonstrated and exploited to efficiently synthesized the target compounds in yields ranging from 81 to 87%. Antiradical activity as well as radical scavenging behavior (H atom-donation, kinetics) of these new fully biobased additives were found superior to that of well-established, commercially available fossil-based antioxidants such as Irganox 1010^®^ and Irganox 1076^®^. Finally, their greater thermal stabilities (302 < T_d_5% < 311 °C), established using thermal gravimetric analysis, combined with their high solubilities and antioxidant activities, make these novel sustainable phenolics a very attractive alternative to current fossil-based antioxidant additives in polyolefins.

## 1. Introduction

In contact with atmospheric oxygen, polymers undergo oxidative degradation reactions during fabrication processes, storage, and throughout their use [1,2,3,4]. Involving undesirable radical species (R^•^) and mechanisms, these oxidative degradation reactions drastically impact the initial aesthetic and mechanical properties of the polymers [1,5,6]. This ineluctable phenomenon is known as thermo-oxidative ageing of polymers. Mixing polymeric materials with a complex blend of additives able to inhibit or delay their deterioration is the best way to prevent premature ageing [7]. These additives are named stabilizers, or more commonly antioxidants. They are classified according to their mechanisms of action: primary (AO-I) and secondary (AO-II) antioxidants.

Sterically-hindered phenols (SHP) such as Irganox 1010^®^ and Irganox 1076^®^, derived from the controversial butylated hydroxytoluene moiety, are the best benchmark antioxidant additives belonging to the AO-I group [7]. They bring oxidative degradation reactions to a close by transferring hydrogen atoms from their phenols to R^•^, resulting in nonreactive phenoxyl radicals. Precisely, SHP act like radical scavengers thanks to chain-breaking reactions, and thus prevent the formation of R^•^, well known to be responsible for polymer degradation [8,9].

Recently, the additive industry has shown increasing interest in naturally occurring *p*-hydroxycinnamic acids (HCAs), such as ferulic, caffeic, sinapic, and *p*-coumaric acids, because of their nontoxic nature yet powerful chain-breaking antioxidative properties, acting through radical scavenging [10,11,12,13,14]. Consequently, we previously reported a library of bisphenolic AO-I derived from HCAs found in lignocellulose (*p*-coumaric, ferulic, and sinapic acids) and biobased diols (1,3-propanediaol, 1,4-butanediol, and isosorbide) [15]. To optimize their antiradical activities, we assessed the structure–activity relationships (aka SAR) of these phenolics [16]. The best candidates, obtained from the reaction between ferulic acid and 1,4-butanediol (BDF), 1,3-propane-diol (PDF), or isosorbide (IDF), exhibited potent antioxidant activities competing with commercially available molecules such as Irganox 1010^®^. Their high thermal stabilities (>200 °C) render them compatible with harsh polymer production processes. Furthermore, their molar mass greater than 500 g.mol^−1^ prevents leaching and other volatility issues. Finally, their eco-friendly preparation makes them an attractive choice as sustainable additives and therefore increases their market value even further. Unfortunately, the polar character of this series of biobased antioxidants leads to a very poor solubility in nonpolar media, such as polyolefins [15]. This major drawback thus limits their reactivity with radicals responsible for oxidation and subsequently reduces their protective role against premature polymer ageing [17]. Indeed, good solubility and mobility have an important positive impact on the stabilization properties of antioxidants [3].

A promising approach to increase compatibility with nonpolar matrix, namely lipophilization, consists in the covalent grafting of lipophilic moieties to phenolics in order to improve both miscibility and incorporation of a given antioxidant in nonpolar media. Lecomte et al. have reported that the grafting of a medium chain-length is the best strategy to design potent lipophilized antioxidants [18]. To date, many of these antioxidants, called phenolipids, have been synthesized from phenolic acid, flavonoids or tocopherols [19,20,21]. However, the design of these molecules is usually not optimized to provide sustainable alternatives able to challenge commercial additives for polymeric materials in terms of activity, solubility and thermal stability.

This work reports on the design and preparation of new potent sustainable bisphenolics AO-I from lignocellulosic and oleaginous biomasses as we dedicate ourselves to integrated biorefinery concepts. To insure a high antiradical activity, the targeted bisphenolic structures were based on our previously reported structure–activity relationship study (SAR) [15,16] and their solubilities into common polymers were evaluated using Hansen and Hildebrand parameters [22]. Once the structural designs were validated, the chemo-enzymatic synthesis of the target structures was optimized through sustainable chemo-enzymatic processes involving a lipase-catalyzed transesterification strategy [23]. Finally, their activities and thermal stabilities were benchmarked against that of Irganox 1010^®^ and lipophilic Irganox 1076^®^, two widely used fossil-based antioxidant additives.

## 2. Experimental

### 2.1. Materials

Ferulic acid, lauric acid, palmitic acid, stearic acid, glycerol, benzyl bromide, *N*,*N*-dimethyl-4-aminopyridine (DMAP), and *N*,*N*-diispropylcarbodiimide (DIC) were purchased from Sigma-Aldrich (Saint-Louis, MO, USA). *Candida antarctica* Lipase B immobilized on resin (LC200291, 10,000 propyl laurate units g^−1^) was obtained from Novozyme. Reagents were used as received. All solvents were bought either from ThermoFisher Scientific (Waltham, MA, USA) or VWR France (Fontenay-sous-Bois, France). Deuterated chloroform (CDCl_3_) was purchased from Euriso-top (Saint-Aubin, France).

### 2.2. Analytical Methods

Column chromatography was carried out with an automated flash chromatograph (PuriFlash 4100, Interchim (Montluçon, France), prepacked with INTERCHIM PF-30SI-HP (30 µm silica gel) columns using a gradient of cyclohexane and ethyl acetate for elution. NMR analyses were recorded on a Bruker (Billerica, MA, USA) Fourier 300. ^1^H NMR spectra of samples were determined in CDCl_3_ at 300 MHz and chemical shifts were reported in parts per million (CDCl_3_, CHCl_3_ residual signal at δ = 7.26 ppm). ^13^C NMR spectra of samples were recorded at 75 MHz (CDCl_3_ signal at δ = 77.16 ppm). High-resolution mass spectroscopy (HRMS) analyses were carried out by the PLANET platform at URCA using a Micromass GC-TOF. Thermogravimetric analyses (TGA) were executed on a Q500 (TA Instruments (Milford, MA, USA)). About 10 mg of each sample was heated from 30 to 500 °C at a rate of 10 °C min^−1^ under constant nitrogen flow (60 mL min^−1^).

### 2.3. Synthesis of Benzylated Ethyl Ferulate

**Figure 1:**
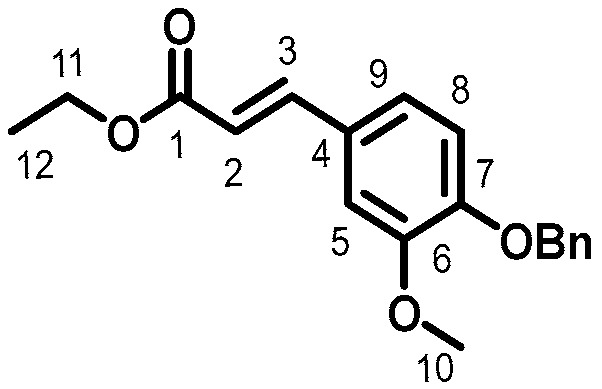
Benzylated ethyl ferulate.

Ethyl ferulate^23^ (25 g, 0.1 mol, 1 eq), benzyl bromide (15 mL, 0.12 mol, 1.2 eq), and K_2_CO_3_ (27 g, 0.2 mol, 2 eq) were dissolved in *N*,*N*-dimethylformamide (DMF) (0.5 M) and heated to 85 °C. The reaction was monitored by TLC and let run until complete conversion of the starting material (3 h). After cooling to room temperature (r.t.), the mixture was concentrated and filtered to remove K_2_CO_3_. The resulting phase was evaporated under reduced pressure and the crude product was purified by flash chromatography on silica gel using cyclohexane and ethyl acetate (90:10) as eluent, providing the desired product as a white powder (32 g, 92%, Figure 1). M.p.: 70.4 °C, ^1^H (300 MHz, CDCl_3_) δ: 3.90 (3H, s, H_10_), 5.18 (2H, s, H_CH2OBn_), 6.30 (2H, d, J = 15.9 Hz, H_2_), 6.83 to 6.87 (3H, m, H_5, 8 and 9_), 7.30 to 7.35 (5H, m, H_ArOBn_), 7.62 (2H, d, J = 15.9 Hz, H_3_), ^13^C (75 MHz, CDCl_3_) δ: 14.4 (C_12_), 56.0 (C_10_), 60.4 (C_11_), 70.8 (C_(CH2)OBn_), 110.2 (C_9_), 113.3 (C_8_), 115.0 (C_2_), 122.8 (C_5_), 128.1 (C_4_), 127.3 to 136. 6 (C_(ArOBn)_), 145.8 (C_7_), 149.8 (C_6_), 150.5 (C_3_), 167.4 (C_1_). 

### 2.4. Lipase-Catalyzed Transesterification of Benzylated Ethyl Ferulate into Glycerol Dibenzyl Ferulate (GDFoBn)

**Figure 2:**
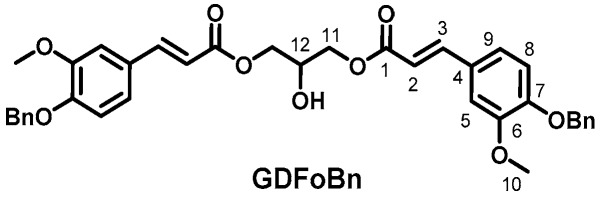
Glycerol dibenzyl ferulate.

Selective lipase-catalyzed transesterification was performed in presence of glycerol (5 g, 54.3 mmol, 1 eq), benzylated ethyl ferulate (42 g, 135.7 mmol, 2.5 eq) and CAL-B (10% *w*/*w* relative to the total weight of batch). The reaction mixture was heated to 75 °C, kept under reduced pressure and magnetically stirred for three days. It was then dissolved in acetone and filtered to remove CAL-B beads. The solvent was evaporated under vacuum and the crude product was purified by flash chromatography on silica gel eluted with cyclohexane/ethyl acetate to provide GDFoBn as a highly viscous oil (32 g, 93%, Figure 2). ^1^H (300 MHz, CDCl_3_) δ: 3.89 (6H, s, H_10_), 4.22 to 4.39 (4H, m, H_11_), 4.28 (1H, s, H_OH_), 5.17 (4H, s, H_(CH2)OBn_), 6.30 (2H, d, J = 15.9 Hz, H_2_), 6.83 to 7.05 (6H, m, H_5, 8 and 9_), 7.25 to 7.40 (10H, m, H_ArOBn_), 7.61 (2H, d, J = 15.9 Hz, H_3_), ^13^C (75 MHz, CDCl_3_) δ: 56.0 (C_10_), 65.4 (C_11_), 68.6 (C_12_), 70.8 (C_(CH2)OBn_), 110.2 (C_9_), 113.3 (C_8_), 115.0 (C_2_), 122.8 (C_5_), 128.14 (C_4_), 127.3 to 136. 6 (C_(ArOBn)_), 145.8 (C_7_), 149.8 (C_6_), 150.5 (C_3_), 167.4 (C_1_).

### 2.5. Lipophilization: Synthesis of GDF_x_

GDFoBn (20 g, 32.1 mmol, 1 eq) and fatty acid (lauric, palmitic or stearic, 1 eq) were dissolved in dichloromethane (DCM) (0.25 M) with a catalytic amount of DMAP, (1.1 g, 9.6 mmol, 0.3 eq). Subsequently, DIC (5.45 mL, 35.2 mmol, 1.1 eq) was added to the mixture and the reaction was magnetically stirred at r.t. overnight. The precipitate urea was removed via filtration and the filtrate concentrated under vacuum. The crude product was dissolved in THF and stirred under N_2_ flow at room temperature. After 10 min, palladium on activated charcoal (Pd/C, 10% *w*/*w*) was added and the solution was stirred under N_2_ for another 10 min, before being submitted to H_2_ flow to simultaneous reduce the C=C double bond and the benzyl protecting group (Bn). The solution was finally filtered using Celite^®^ pads and evaporated under reduced pressure. Target bisphenol was purified by flash chromatography on silica gel eluted with cyclohexane/ethyl acetate. Structures were named GDF_x,_ for Glycerol Diferulate, where the incrementation “x” indicates the alkyl chain length.

**Figure 3:**
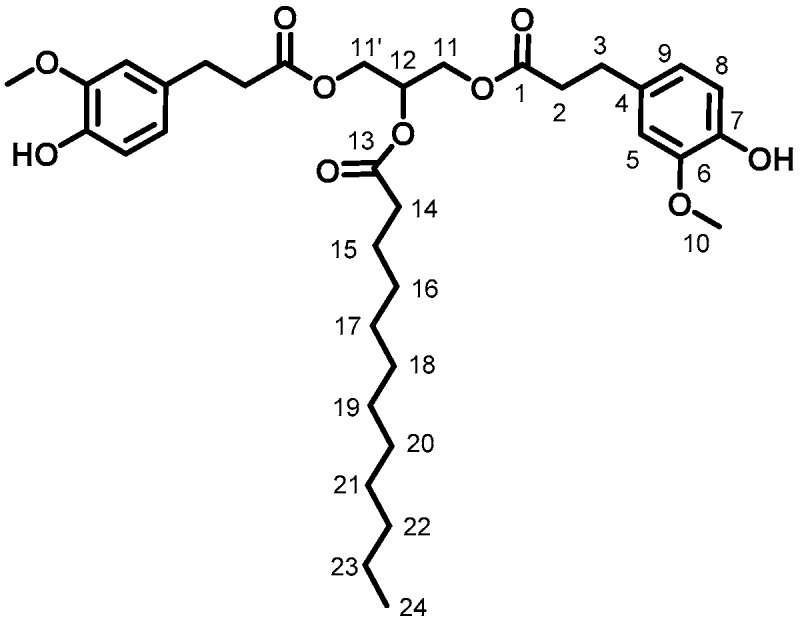
GDF_10_.

GDF_10_ (84%, Figure 3). ^1^H (300 MHz, CDCl_3_) δ: 0.87 (3H, t_app_, J = 6.8 Hz, H_24_), 1.24 (16H, m, H_16 to 23_), 1.61 (2H, m, H_15_), 2.28 (2H, t, J = 7.5 Hz, H_14_), 2.60 (4H, t, J = 7.6 Hz, H_3_), 2.86 (4H, t, J = 7.6 Hz, H_2_), 3.79 (6H, s, H_10_), 4.18 (4H, dd, J = 4.2, 11.7 Hz, H_11, 11′_), 5.22 (1H, m, H_12_), 5.53 (2H, s, H_OH_), 6.65 (2H, s, H_5_), 6.68 (2H, s, H_9_), 6.81 (2H, d, J = 7.8 Hz, H_8_), ^13^C (75 MHz, CDCl_3_) δ: 14.2 (C_24_), 24.9 (C_15_), 29.1–29.7 (C_16 to 23_), 30.6 (C_3_), 34.2 (C_14_), 36.1 (C_2_), 55.9 (C_10_), 62.3 (C_11_), 68.8 (C_12_), 110.9 (C_9_), 114.5 (C_8_), 120.9 (C_5_), 132.2 (C_4_), 144.2 (C_7_), 146.5 (C_6_), 172.5 (C_1_), 173.0 (C_13_), FT-IR (neat), ν_max_: 3456 (ArOH), 2922 and 2851 (aliphatic chain), 1733 (C=O), Td_5%-Loss_: 302 °C, HRMS (TOF MS, ES+): *m*/*z* calcd for C_35_H_50_O_10_Na: 653.3295; found 653.3302. 

**Figure 4:**
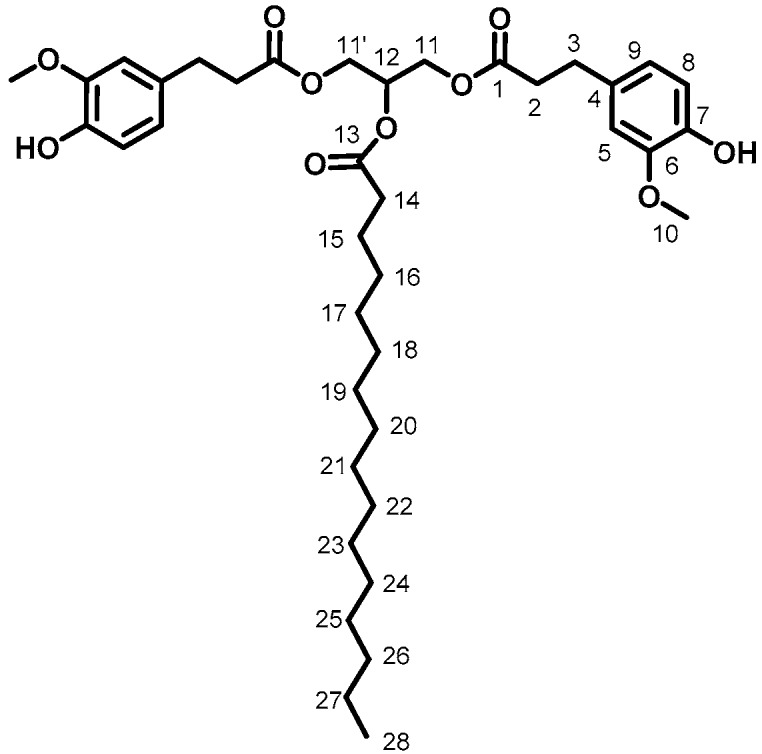
GDF_14_.

GDF_14_ (81%, Figure 4). ^1^H (300 MHz, CDCl_3_) δ: 0.87 (3H, t_app_, J = 6.3 Hz, H_28_), 1.23 (24H, m, H_16 to 27_), 1.59 (2H, m, H_15_), 2.28 (2H, t, J = 7.5 Hz, H_14_), 2.61 (4H, t, J = 5.1 Hz, H_3_), 2.88 (4H, t, J = 7.5 Hz, H_2_), 3.78 (6H, s, H_10_), 4.17 (4H, dd, J = 4.5, 12.0 Hz, H_11, 11′_), 5.23 (1H, m, H_12_), 5.51 (2H, s, H_OH_), 6.65 (2H, s, H_5_), 6.68 (2H, s, H_9_), 6.81 (2H, d, J = 7.8 Hz, H_8_), ^13^C (75 MHz, CDCl_3_) δ: 14.2 (C_28_), 24.9 (C_15_), 29.1–29.7 (C_16 to 27_), 30.6 (C_3_), 34.2 (C_14_), 36.0 (C_2_), 55.9 (C_10_), 62.3 (C_11_), 68.8 (C_12_), 110.9 (C_9_), 114.5 (C_8_), 120.9 (C_5_), 132.2 (C_4_), 144.2 (C_7_), 146.5 (C_6_), 172.5 (C_1_), 173.0 (C_13_), FT-IR (neat), ν_max_: 3460 (ArOH), 2921 and 2850 (aliphatic chain), 1734 (C=O), Td_5%-Loss_: 311 °C, HRMS (TOF MS, ES+): *m*/*z* calcd for C_39_H_58_O_10_Na: 709.3937; found 709.3928. 

**Figure 5:**
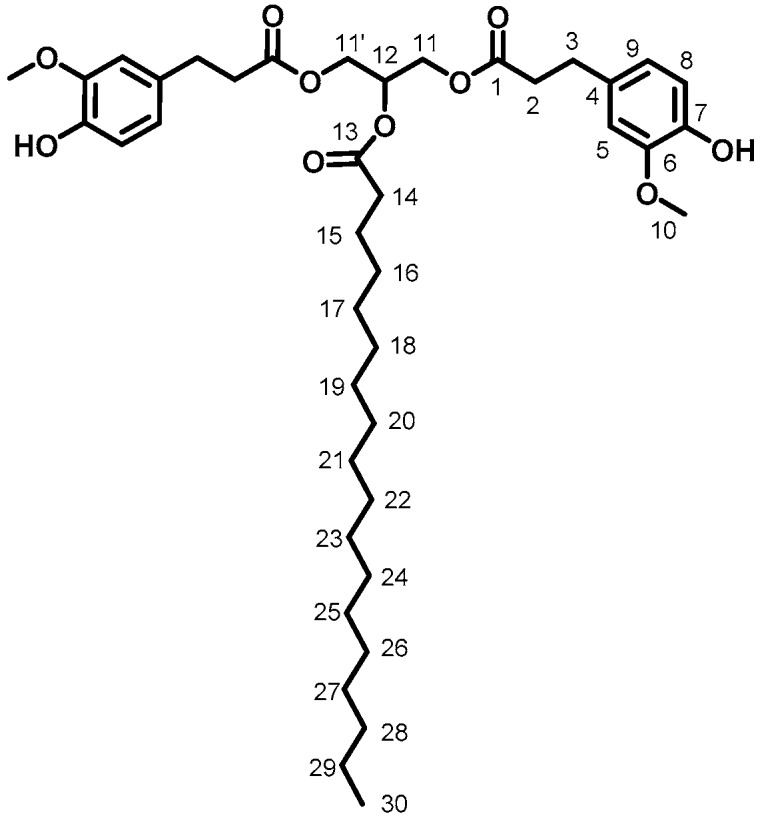
GDF_16_.

GDF_16_ (87%, Figure 5). ^1^H (300 MHz, CDCl_3_) δ: 0.87 (3H, t_app_, J = 6.3 Hz, H_30_), 1.24 (28H, m, H_16 to 30_), 1.58 (2H, m, H_15_), 2.27 (2H, t, J = 7.5 Hz, H_14_), 2.60 (4H, t, J = 8.1 Hz, H_3_), 2.85 (4H, t, J = 7.5 Hz, H_2_), 3.86 (6H, s, H_10_), 4.17 (4H, dd, J = 4.5, 12.0 Hz, H_11, 11′_), 5.23 (1H, m, H_12_), 5.48 (2H, s, H_OH_), 6.65 (2H, s, H_5_), 6.68 (2H, s, H_9_), 6.81 (2H, d, J = 7.5 Hz, H_8_), ^13^C (75 MHz, CDCl_3_) δ: 14.2 (C_30_), 24.9 (C_15_), 27.1–29.7 (C_16 to 29_), 30.6 (C_3_), 34.2 (C_14_), 36.0 (C_2_), 55.9 (C_10_), 62.3 (C_11_), 68.8 (C_12_), 110.9 (C_9_), 114.5 (C_8_), 120.9 (C_5_), 132.2 (C_4_), 144.2 (C_7_), 146.5 (C_6_), 172.5 (C_1_), 173.0 (C_13_), FT-IR (neat), ν_max_: 3454 (ArOH), 2921 and 2850 (aliphatic chain), 1735 (C=O), Td_5%-Loss_: 308 °C, HRMS (TOF MS, ES+): *m*/*z* calcd for C_41_H_62_O_10_Na: 737.4252; found 737.4241.

### 2.6. Calculation of Solubility Parameters

The theoretical solubility and incorporation of additives into polymers were estimated by using the van Krevelen and Hoftyzer atomic group contribution method [22]. The Hansen solubility parameters of compounds were calculated using following equation and the database supplied in electronic supplementary information (ESI).

δ_d_ = ΣF_di_/ΣV_i_; Dispersion component (J^1/2^/cm^−3/2^) (1)

δ_p_ = ΣF²_pi_/ΣV_i_; Polar component (J^1/2^/cm^−3/2^) (2)

δ_h_ = ΣE_hi_/ΣV_i_; Hydrogen-bonding component; (J^1/2^/cm^−3/2^) (3)

with

F_di_: Dispersion contribution of the molar attraction constant [(J^1/2^ cm^−3/2^)/mol^−1^]
F_pi_: Polar contribution of the molar attraction constant [(J^1/2^ cm^−3/2^)/mol^−1^]
E_hi_: Hydrogen-bonding energy contribution of the molar attraction constant (J/mol)
V: Molar volume contribution of the chemical group involved (cm^3^/mol).

The Hildebrand solubility parameters (HiSP) were then calculated in J^1/2^/cm^−3/2^ with the simplified following equation:

HiSP = √ (δ²_d_ + δ²_p_ + δ²_h_) (4)

### 2.7. Analysis of the Radical Scavenging Power of Antioxidants

The radical scavenging power was measured with a method derived from Berset et al. [24]. A total amount of 190 µL homogenate DPPH (2,2-diphenyl-1-picrylhydrazyl) ethanol solution (200 µM) was added to a 96-well plate containing 10 µL of potential antiradical molecule ethanol solutions at different concentrations, ranging from 300 µM to 9.3 µM. The experiments were performed in an invariable excess of the DPPH radical (200 µM, 40 nmol). The phenolic concentrations were selected to get linear dose-response intervals. The reaction was followed by a microplate Multiskan FC 1 scan every 5 min for 7.5 h at 515 nm. The use of different amounts of the potential antioxidant gave rise to the EC_50_ value, which is defined as the concentration needed to reduce half the initial amount of DPPH. Each analysis was performed four times. The total stoichiometries (n) is the number of DPPH moles reduced by 1 mol of antioxidant (*n* = DPPH_tot_/EC_50_ × 2).

## 3. Results and Discussions

### 3.1. Design of Lipophilic Antioxidants: Predictive Approaches

**Figure 6:**
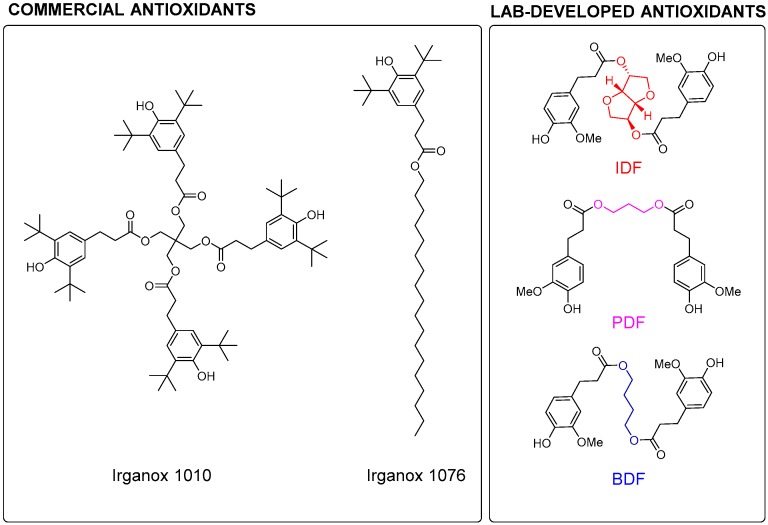
Developed structures of phenolic additives.

The design of the new bisphenolic antioxidants was based on our earlier studies on the Structure–Activity Relationships (SAR) of ferulic acid-based diphenolic antioxidants [15]. Ferulic acid is a well-known natural antioxidant because of its hydrogen-donating ability [13,25]. It was shown that removing the α,β-unsaturation was beneficial for the radical-scavenger ability of bisphenol structures [16]. Therefore, α,β-saturated ferulic acid moieties were selected to confer optimal radical scavenging ability. A previously published SAR study [16] showed that the nature of the linker between the antiradical moieties slightly impacts the antioxidant activity. In this specific study, the linker had to be a triol able to carry the two phenolic moieties as well as an alkyl chain allowing the fine tuning of the lipophilic character of the molecule. Glycerol that carries two primary alcohols and one secondary alcohol was selected. Glycerol is a byproduct during the production of biodiesel, therefore available in high amounts, the use of which helps reduce the environmental impact of this industry [26]. The lipophilic part was based on recent advances, which show that the grafting of a medium chain-length fatty acid can be used to design potent custom-made lipophilic antioxidants [18,21]. Herein, three linear fatty acids were tested. The theoretical miscibility of additives used in the formulation with PP was calculated with the help of the Hildebrand solubility parameters (HiSP, Equation (4)). The lower the difference of HiSP between two compounds, the higher their mutual solubility. In the literature HiSP values in the range between 16.8 and 19.0 J^1/2^ cm^−3/2^ are reported for PP. The bisphenolics previously developed in our laboratory (i.e., BDF, PDF, and IDF) exhibited HiSP values greater than 25.0 J^1/2^/cm^−3/2^ (Figure 6, Table 1). Among them, IDF has the highest polarity contribution (δ_p_ 7.2 vs. 3.5–3.7 J^1/2^/cm^−3/2^) linked to the polarity of the isosorbide linker. In comparison, benchmark antioxidant Irganox 1010^®^ exhibits a low HiSP value of 21.4 J^1/2^/cm^−3/2^. The commercial lipophilic antioxidant Irganox 1076^®^ exhibits a HiSP value much more comparable to that of PP, thanks to a lower contribution of the aliphatic chain to polarity (δ_p_) and the other hydrogen bonding (δ_h_) factors [27]. The increase of the polar contribution of the target antioxidants was calculated for esters with lauric (C_12_), palmitic (C_16_), and stearic (C_18_) acid. This group of lipophilic antioxidants was named GDF_x,_ for Glycerol Diferulate, where the incrementation “x” indicates the alkyl chain length. The HiSP values of the GDF_x_ family were lowered compared to the initial antioxidants, therefore better solubility in PP might be expected (Table 1). The HiSP values showed the importance of alkyl chain length. Indeed, data show that an increase of the chain length by six carbon atoms (GDF_10_ vs. GDF_16_) leads to a HiSP value drop of 1.00 J^1/2^/cm^−3/2^ (Table 1).

### 3.2. Synthesis of the Targets (GDF_x_)

**Figure 7:**
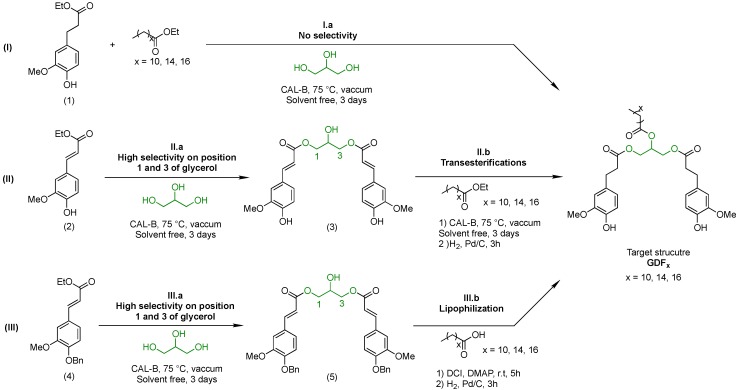
Stoichiometric one pot-one step enzymatic strategy (**I**), one pot-two step strategy (**II**), chemo-enzymatic strategy (**III**), and corresponding yields (I.a: 34%, II.a: 91%, II.b: 40%, III.a: 93%, III.b GDF_10_: 84%, GDF_14_: 81%, GDF_16_: 87%).

As we committed ourselves to the use of sustainable processes for the production of the targeted bisphenols, the use of the previously reported lipase-catalyzed synthesis of IDF, PDF, and BDF was envisaged [23]. Indeed, the use of immobilized lipase CAL-B as a biocatalyst is a great tool for the development of sustainable processes. Not only does CAL-B not require a solvent, it is inactive toward phenolic hydroxyl groups, but can also be reused for further reaction cycles. In addition, CAL-B has been proven to promote the easier (trans)esterification on esters rather than acids moieties. Hence, α,β-saturated ethyl ferulate (aka ethyl dihydroferulate) and fatty acid ethyl ester (FAEE) were selected as starting materials rather than the corresponding acids. Several strategies were investigated to determine the best synthetic pathway to GDF_x_. The different pathways (Figure 7) are the

(I) Stoichiometric one pot-one step enzymatic strategy,
(II) One pot-two step strategy, and
(III) Chemo-enzymatic strategy.

### 3.3. Stochiometric One Pot-One Step Enzymatic Strategy (Pathway I, Figure 7)

The first strategy (I) involved a one pot-one step lipase-mediated transesterification of glycerol with a mixture of ethyl dihydroferulate (1) (2 eq) and FAEE (1 eq) (I.a—Figure 7). It is worth mentioning that ethyl dihydroferulate can be easily obtained from ethyl ferulate through a simple palladium-catalyzed hydrogenation in yield greater than 98% [23]. Under such stoichiometric conditions, *Candida antarctica* lipase B was not regioselective toward glycerol primary and secondary alcohols. Indeed, despite the total conversion of ethyl dihydroferulate, the enzyme functionalized primary and secondary alcohols randomly with ethyl dihydroferulate or FAEE, leading to a complex mixture of the target structure and corresponding regioisomers. After purification, the targeted GDF_x_ were isolated in 34% yield.

### 3.4. One Pot-Two Step Strategy

The transesterification was then performed on α,β-unsaturated ethyl ferulate (2). In that case, *Candida antarctica* lipase B proved unable to esterify the secondary alcohol of glycerol, giving access to the symmetric bisphenol (3) in high yield after purification (II.a, 91%, Figure 7). The α,β-unsaturation of ethyl ferulate (2) conferred probably more rigidity to the molecule and decreased the accessibility to the active site of the lipase. Furthermore, it also decreased significantly the electronic density on the C from the carbonyl, thus limiting its reactivity. With the aim to access GDF_x_, a second step involving a lipase-catalyzed transesterification followed by a palladium-catalyzed hydrogenation was then performed on intermediate bisphenol (3) (II.b, Figure 7). This second biocatalytic step induced however transesterification issues due to CAL-B esterase activity, resulting in a complex mixture of regioisomers and therefore in the decrease in yield down to 40% after purification.

### 3.5. Chemo-Enzymatic Strategy

A protecting group-based chemo-enzymatic strategy was finally developed to produce lipophilic antioxidants in high yields. The first step involved the lipase-mediated transesterification of benzylated ethyl ferulate (4) with the two primary alcohols of glycerol leading to an intermediate bisphenol (5) isolated in 93% yield (III.a, Figure 7). Then, fatty acids were grafted onto the available secondary alcohol through a conventional Steglich esterification, immediately followed by a palladium-catalyzed hydrogenation to simultaneously reduce the α,β-unsaturation and cleave the benzyl protecting group (III.b, Figure 7). Using this third strategy, targeted bisphenols were obtained as single regioisomers in high yields (GDF_10_: 84%, GDF_14_: 81%, GDF_16_: 87%). It is worth mentioning that this synthesis was then successfully implemented on a large scale (≈20 g).

The thermal properties of the new additives described in Table 2 were investigated by thermogravimetric analysis (TGA).

Thermogravimetric analyses (TGA) of the bisphenols revealed thermostability (T_d_5%) in the range of 302 to 311 °C. Furthermore, the alkyl chain length does not significantly impact the degradation temperature. Indeed, replacing lauric (C_12_) with palmitic (C_16_) or stearic (C_18_) moieties shift their thermostability by less than 10 °C.

### 3.6. Analysis of the Antiradical Activity of Lipophilic Bisphenols

The DPPH method is an easy and rapid analytical tool to determine the free radical scavenging activity of phenolic compounds and their abilities to quench radicals. [24] The total H atom donation capacities can be evaluated with the EC_50_ value, defined as the quantity of antioxidant (nmol) needed to protonate/quench 50% of the initial population of DPPH. The lower the EC_50_, the higher the antioxidant activity of a compound is. Because the EC_50_ is intrinsically linked to the structure of the antioxidant, in particular to the number of free phenols, a compound can be also characterized by its antioxidant stoichiometry (n), i.e., the number of DPPH radicals reduced by one molecule of antioxidant [28]:

n = DPPH_tot_/EC_50_ × 2 (5)

where DPPH_tot_ is the initial amount of DPPH in nmol.

The principal kinetic parameters of the DPPH reaction are the time needed to reach the steady state at the concentration EC_50_, i.e., T_EC50_, and the rate of reaction towards free radicals [29]. By combining H atom-donation abilities and kinetic parameters, the Antioxidant Efficiency (AE) [29,30] can be defined in order to characterize the behavior of a substance as antioxidant after:

AE = (1/EC_50_) × ׀m(Ec_50_)׀ (6)

with m(EC_50_) the slope in the first minutes of the absorbance-versus-time plots at EC_50_ of each compound.

**Figure 8:**
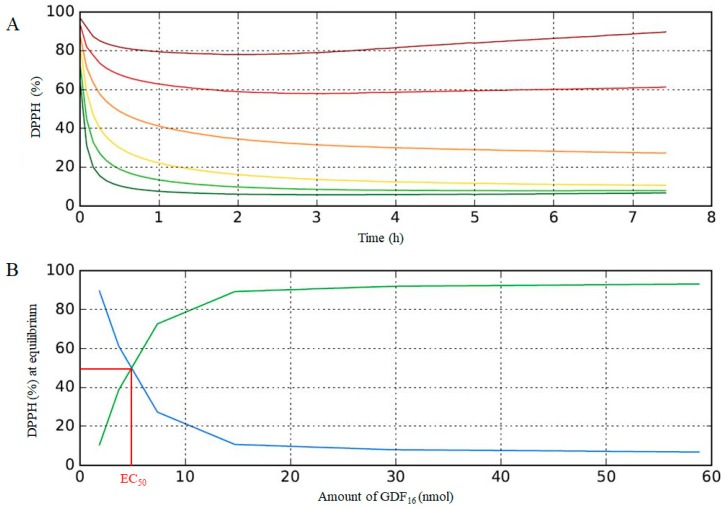
(**A**) Reaction kinetics of GDF_16_ solution at given concentrations: 300 µM (dark green), 150 µM (light green), 75 µM (yellow), 37.5 µM (orange), 18.8 µM (red), and 9.4 µM (brown), (**B**) EC_50_ determination for GDF_16_. Blue line: 2,2-diphenyl-1-picrylhydrazyl (DPPH) radical and Green line: RPPH reduced.

The reaction kinetics of all compounds with DPPH are shown in ESI, and an example of EC_50_ determination can been seen in Figure 8. The reaction proceeds in two stages, with a fast decay in absorbance during the first minutes, followed by a slower one until equilibrium is reached. EC_50_ values and total stoichiometries (n) obtained with the newly synthesized and commercial phenolic additives classified by chemical structure are shown in Table 3.

As shown in Table 3, the EC_50_ values for GDF_x_ are homogenous around 5 nmol (mean value: 4.95 nmol). It is noteworthy to mention that no correlation between the alkyl chains size and the antiradical activity was observed. Irganox 1010^®^ is the most potent scavenging compound, whereas Irganox 1076^®^ is the least one; GDF_x_ have intermediate EC_50_ values. The number of phenols available for DPPH quenching is an important feature for the antioxidant activity. Irganox 1010^®^ and 1076^®^ contains four and one free phenol groups, respectively, while the GDF_x_ family has only two. The stoichiometries value n takes into account this factor. As expected, with its four phenols, Irganox 1010^®^ exhibited an antiradical activity twice as high than that found for the GDF_x_ family that only bears two phenols. Similarly, the monophenolic compound Irganox 1076^®^ displayed an activity twice as low than the bisphenolic compound GDF_x_.

**Figure 9:**
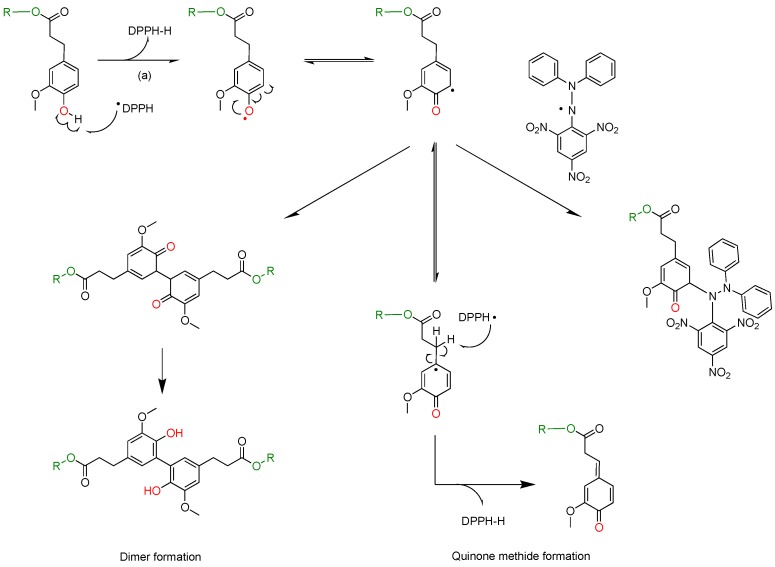
Chemical behavior of phenolic antioxidants.

The stoichiometry value n does not coincide with the number of hydroxyl groups available. Indeed, the direct abstraction of the phenol H-atom and the electron transfer process from ArOH to DPPH radical is not the only mechanism involved in the reaction between DPPH and phenols. Various studies [31,32] have already proposed potential phenol regeneration pathways leading to reaction products such as dimers or quinone methides, able to further react with DPPH radicals and thus leading to n values higher than 2 (Figure 9).

Finally, in order to determine the antioxidant efficiencies (AE), the absorbance-vs-time plots at the EC_50_ concentration of each phenolic compound were established. When concentration of DPPH was in large excess, the slope in the first minutes of these plots (m_EC50_ = Δy/Δx 0→10 min) could be assimilated to the rate constants of fast reaction of proton abstraction (a) in Figure 9 [29]. Consequently, antioxidant efficiency values (AE) calculated using equation (6) (Table 3 and ESI), take into consideration both H atom-donation capacity and kinetic aspects of the phenolic compounds tested. Here, the higher the AE, the higher the antioxidant activity of a compound is.

Based on these results, the antioxidant Irganox 1076^®^ was the least efficient of all tested phenolic compounds. It is interesting to note that a compound such as Irganox 1010^®^, with an EC_50_ value of 2.50, was found to be the best antioxidant of all phenolic compounds, but instead showed a moderate antioxidant efficiency (AE = 0.30), due to its relative slow rate of reaction (Table 3). Finally, the newly created GDF_x_ family appeared to be competitive showing the highest AE values, ranging from 0.42 to 0.46, due to the combination of high H atom-donation capacities and fast kinetics.

## 4. Conclusions

A novel class of renewable bisphenol (GDF_x_) was successfully designed from ferulic acid and vegetal oil (glycerol and fatty acids) and proved to be potent antioxidant additives for polyolefins. By playing with the chemical structure of the starting materials, their polarities can be easily tuned so to display theoretical solubilities into polypropylene similar to that of Irganox 1010^®^. The efficient synthesis of these novel bisphenols has been achieved through a chemo-enzymatic process involving a highly regioselective lipase-mediated transesterification, allowing the preparation of well structurally defined targets in very good yields (81–87%). The H atom-donation capacities and kinetic aspects of these novel bisphenols were evaluated using the DPPH free radical technique and benchmarked against commercially available antioxidant compounds. All of the results demonstrated the high radical scavenging capacity of these renewable bisphenols and their potential as promising renewable alternatives. The study of their antioxidant capacity in polyolefins matrices will be reported in due course.

## Figures and Tables

**Table 1:** Hildebrand solubility parameters (HiSP) of phenolic antioxidants.

Compound	δ_d_ (J^1/2^ cm^−^^3/2^)	δ_p_ (J^1/2^ cm^−^^3/2^)	δ_h_ (J^1/2^ cm^−^^3/2^)	HiSP (J^1/2^ cm^−^^3/2^)
BDF	21.3	3.5	13.6	25.5
PDF	21.5	3.7	13.9	25.9
IDF	23.3	7.2	14.9	28.5
GDF_10_	19.8	2.5	11.7	23.2
GDF_14_	19.5	2.2	10.9	22.5
GDF_16_	19.3	2.1	10.7	22.2
Irganox 1010^®^	18.9	1.3	10.0	21.4
Irganox 1076^®^	17.4	1.3	7.0	18.8

**Table 2:** Thermostability (T_d_5%) of GDF_x_.

Compound	Thermostability (T_d_5%, °C)
GDF_10_	302
GDF_14_	311
GDF_16_	308
Irganox1076^®^	236
Irganox1010^®^	347

**Table 3:** Radical scavenging parameters of phenolic antioxidants.

Compound	Free Phenols	EC_50_ (nmol)	Stoichiometries (n)	׀m(EC_50_)׀	AE
GDF_10_	2	4.81 ± 0.17	4.17 ±0.15	2.19	0.45
GDF_14_	2	5.38 ± 0.12	3.72 ± 0.09	2.30	0.42
GDF_16_	2	4.66 ± 0.15	4.30 ± 0.14	2.16	0.46
Irganox 1010^®^	4	2.52 ± 0.16	7.98 ± 0.47	0.76	0.30
Irganox 1076^®^	1	11.48 ± 0.17	1.74 ± 0.03	0.68	0.06

## Supplementary Materials

The following are available online at http://www.mdpi.com/1422-0067/19/11/3358/s1.
