# Supplementary material for: Chemo-Enzymatic Synthesis of Renewable Sterically-Hindered Phenolic Antioxidants with Tunable Polarity from Lignocellulose and Vegetal Oil Components
Source: Int J Mol Sci. 2018 Oct 26;19(11):3358. doi: 10.3390/ijms19113358 (PMC6274793; doi:10.3390/ijms19113358)

Chemo-enzymatic synthesis of renewable sterically-hindered phenolic antioxidants with tunable polarity from lignocellulose and vegetal oil components

L.; Hollande,[a,b] ; Domenek,[b] S.; Allais,*[a] F.

[a] Chaire ABI, AgroParisTech, CEBB, 3 rue des Rouges Terres 51110 Pomacle, France
[b] UMR GENIAL, AgroParisTech, INRA, Université Paris-Saclay, Avenue des Olympiades, 91300 Massy, France

florent.allais@agroparistech.fr

Electronic Supplementary Information

Table of contents

Molar constant for the calculation of Hansen solubility parameters. 3

^{1}H NMR spectrum of benzylated ethyl ferulate ($CDCl_3$) 4

^{13}C NMR spectrum of benzylated ethyl ferulate ($CDCl_3$) 5

^{1}H NMR spectrum of GDFOBn from lipase catalysed transesterification ($CDCl_3$).. 6

^{13}C NMR spectrum of GDFOBn from lipase catalysed transesterification ($CDCl_3$).. 7

^{1}H NMR spectrum of GDF_{10} ($CDCl_3$) 8

^{13}C NMR spectrum of GDF_{10} ($CDCl_3$) 9

FT-IR spectra of GDF_{10} 10

HRMS analysis of GDF_{10}.. 11

TGA analysis of GDF_{10} 12

DPPH analysis (EC_{50}) of GDF_{10}.. 13

^{1}H NMR spectrum of GDF_{14} ($CDCl_3$) 14

^{13}C NMR spectrum of GDF_{14} ($CDCl_3$) 15

FT-IR spectra of GDF_{14} 16

HRMS analysis of GDF_{14}..17

TGA analysis of GDF_{14}...18

DPPH analysis (EC_{50}) of GDF_{14}..19

^{1}H NMR spectrum of GDF_{16} ($CDCl_3$) 20

^{13}C NMR spectrum of GDF_{16} ($CDCl_3$) 21

FT-IR spectra of GDF_{16} 22

HRMS analysis of GDF_{16}..23

TGA analysis of GDF_{16}...24

DPPH analysis (EC_{50}) of GDF_{16}..25

Kinetics behaviours at EC_{50} concentration for GDF_{10}, GDF_{14}, GDF_{16}, $Irganox_{1010}$, Irganox $_{1076}$ 26

Molar constant for the calculation of Hansen solubility parameters.

Structural group	F_d ($J^{1/2}$ $cm^{-3/2}$ mol^{-1})	F_p^2 (J cm^{-3} mol^{-1})	E_h (J/mol)	V (cm^3 mol^{-1})
$-CH_3$	420	0	0	33.5
$-CH_2-$	270	0	0	16.1
-CH-	80	0	0	-1
>C<	-70	0	0	-19.2
Phenyl (trisubstituted)	1270	12100	0	33.4
-OH	210	250000	20000	10
-O-	100	160000	3000	3.8
-COO-	390	240100	7000	18
Plane of symmetry	/	Total x0.5 if 1<br>Total x0.25 if 2<br>Total x0 if 3 or more	Total x0 if 3 or more	/

F_d : dispersion contribution; F_p : polar contribution; E_h: hydrogen-bonding-energy contribution; V : molar volume

^{1}H NMR spectrum of benzylated ethyl ferulate ($CDCl_3$)

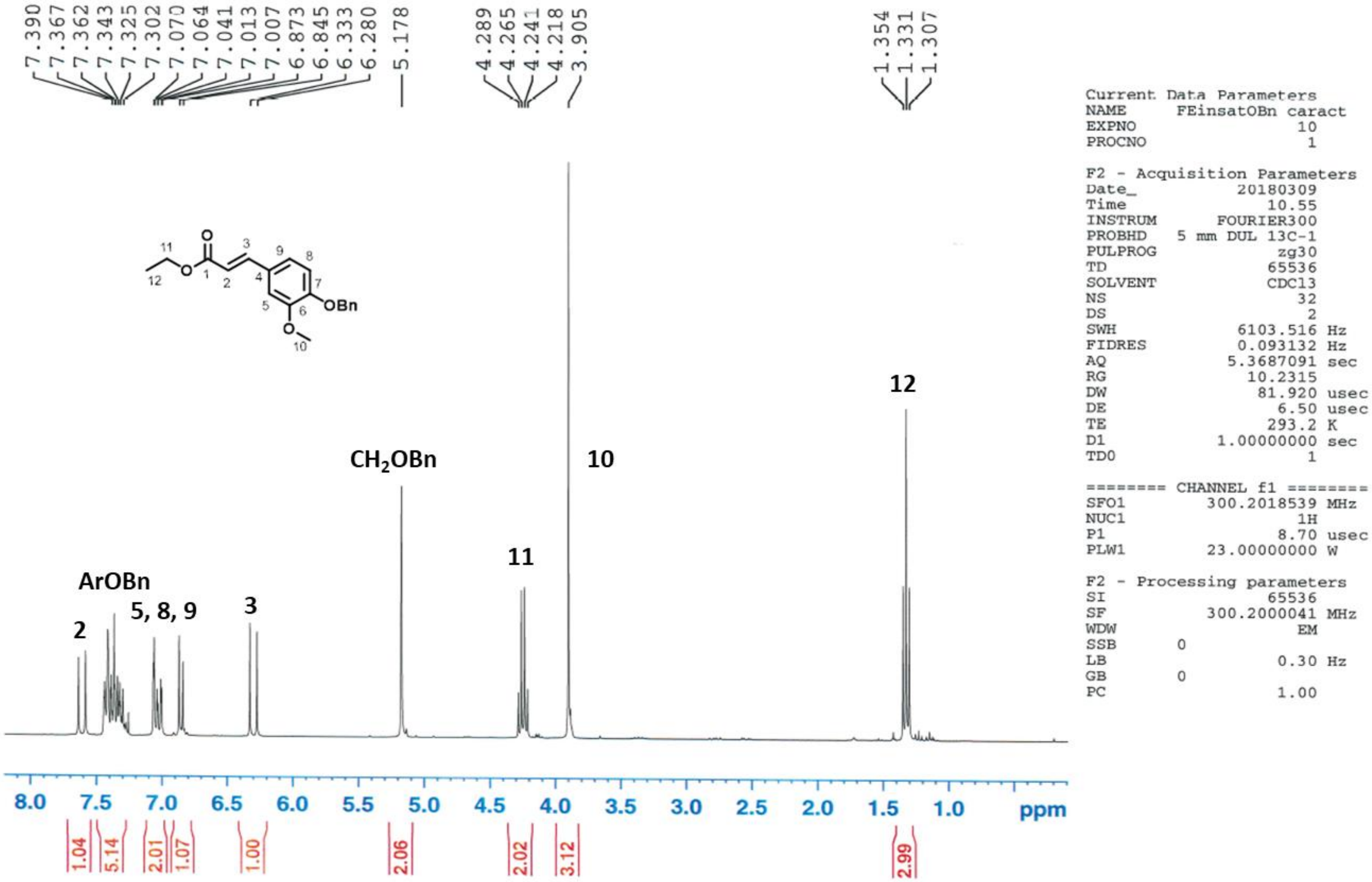

^{13}C NMR spectrum of benzylated ethyl ferulate ($CDCl_3$)

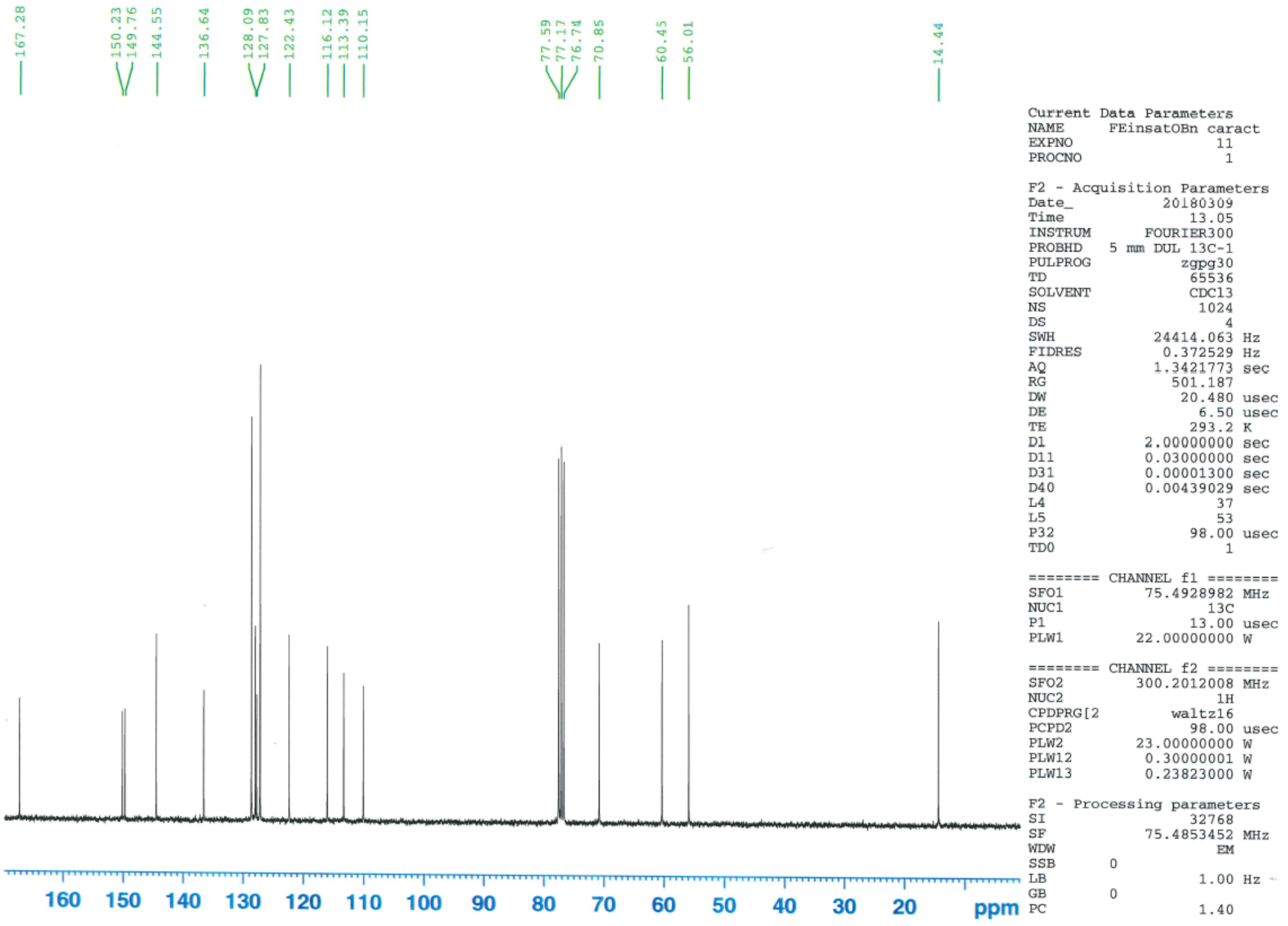

^{1}H NMR spectrum of GDFOBn from lipase catalysed transesterification ($CDCl_3$)

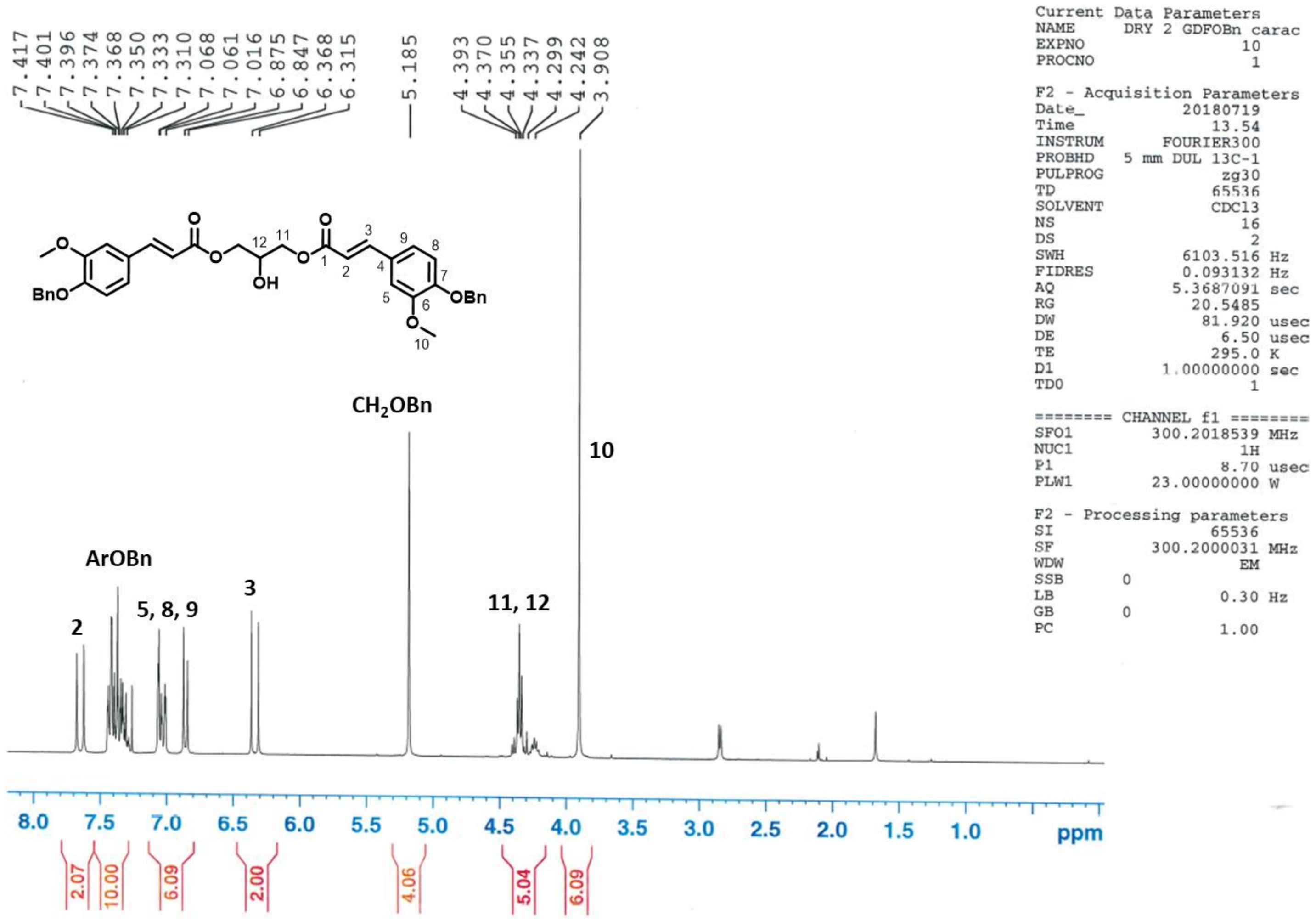

^{13}C NMR spectrum of GDFOBn from lipase catalysed transesterification ($CDCl_3$)

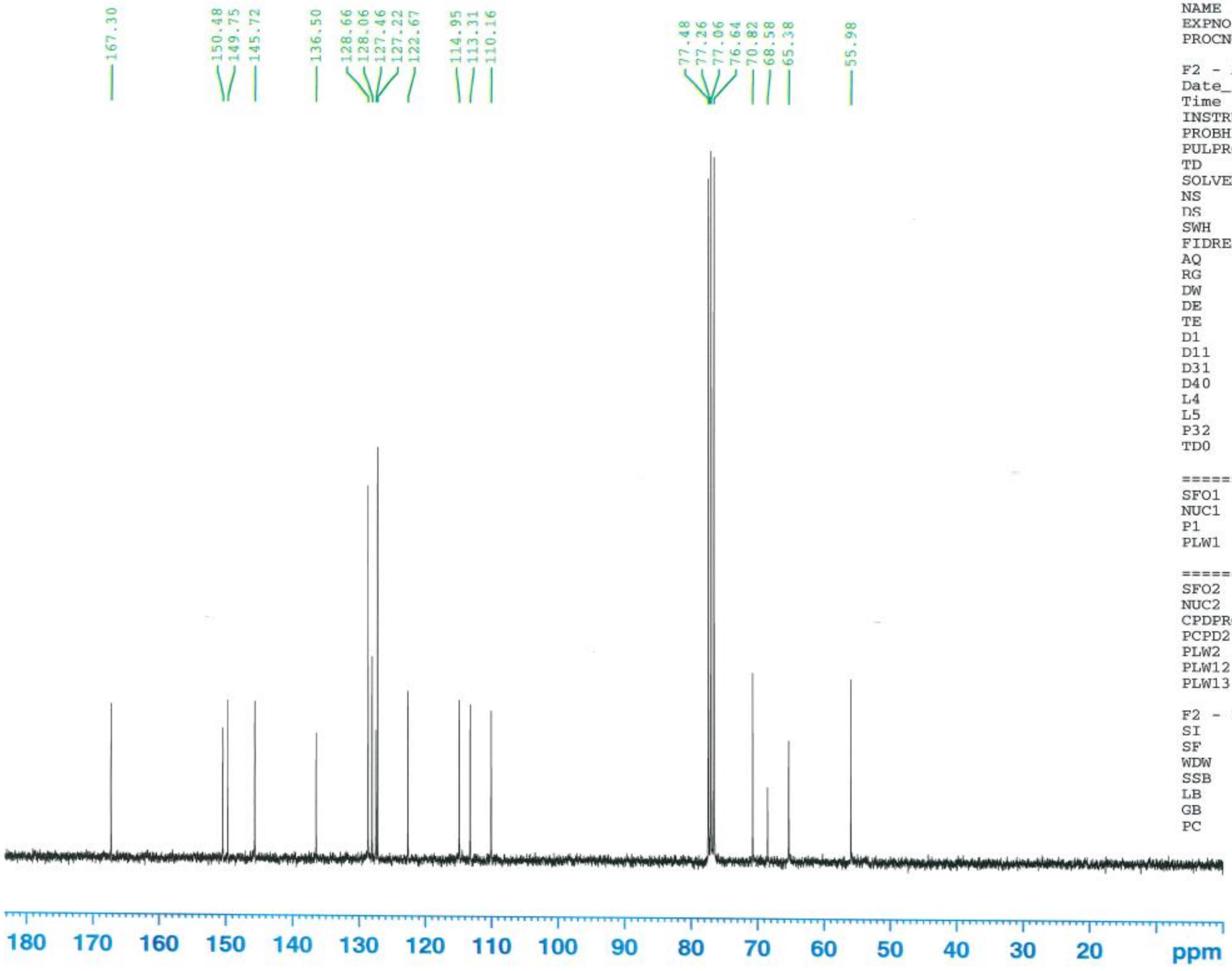

Current Data Parameters
NAME DRY 2 GDFOBn carac
EXPNO 11
PROCNO 1

F2 - Acquisition Parameters
Date_ 20180719
Time 14.03
INSTRUM FOURIER300
PROBHD 5 mm DUL 13C-1
PULPROG zgpg30
TD 65536
SOLVENT CDCl3
NS 1024
DS 4
SWH 24414.063 Hz
FIDRES 0.372529 Hz
AQ 1.3421773 sec
RG 501.187
DW 20.480 usec
DE 6.50 usec
TE 295.2 K
D1 2.00000000 sec
D11 0.03000000 sec
D31 0.00001300 sec
D40 0.00439029 sec
L4 37
L5 53
P32 98.00 usec
TD0 1

======== CHANNEL f1 ========
SFO1 75.4928982 MHz
NUC1 13C
P1 13.00 usec
PLW1 22.00000000 W

======== CHANNEL f2 ========
SFO2 300.2012008 MHz
NUC2 1H
CPDPRG[2 waltz16
PCPD2 98.00 usec
PLW2 23.00000000 W
PLW12 0.30000001 W
PLW13 0.23823000 W

F2 - Processing parameters
SI 32768
SF 75.4853500 MHz
WDW EM
SSB 0
LB 1.00 Hz
GB 0
PC 1.40

^{1}H NMR spectrum of GDF_{10} ($CDCl_3$)

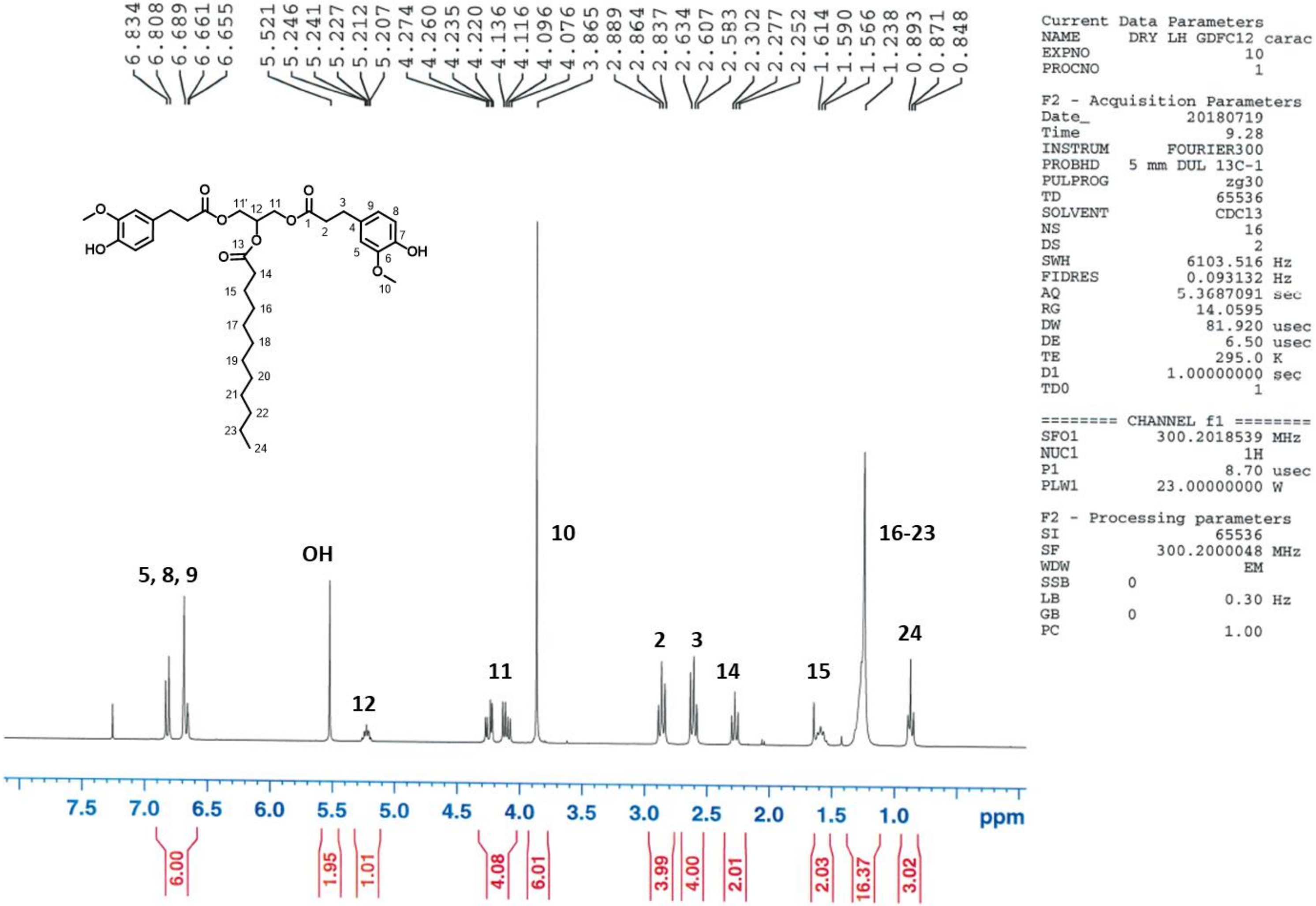

^{13}C NMR spectrum of GDF_{10} ($CDCl_3$)

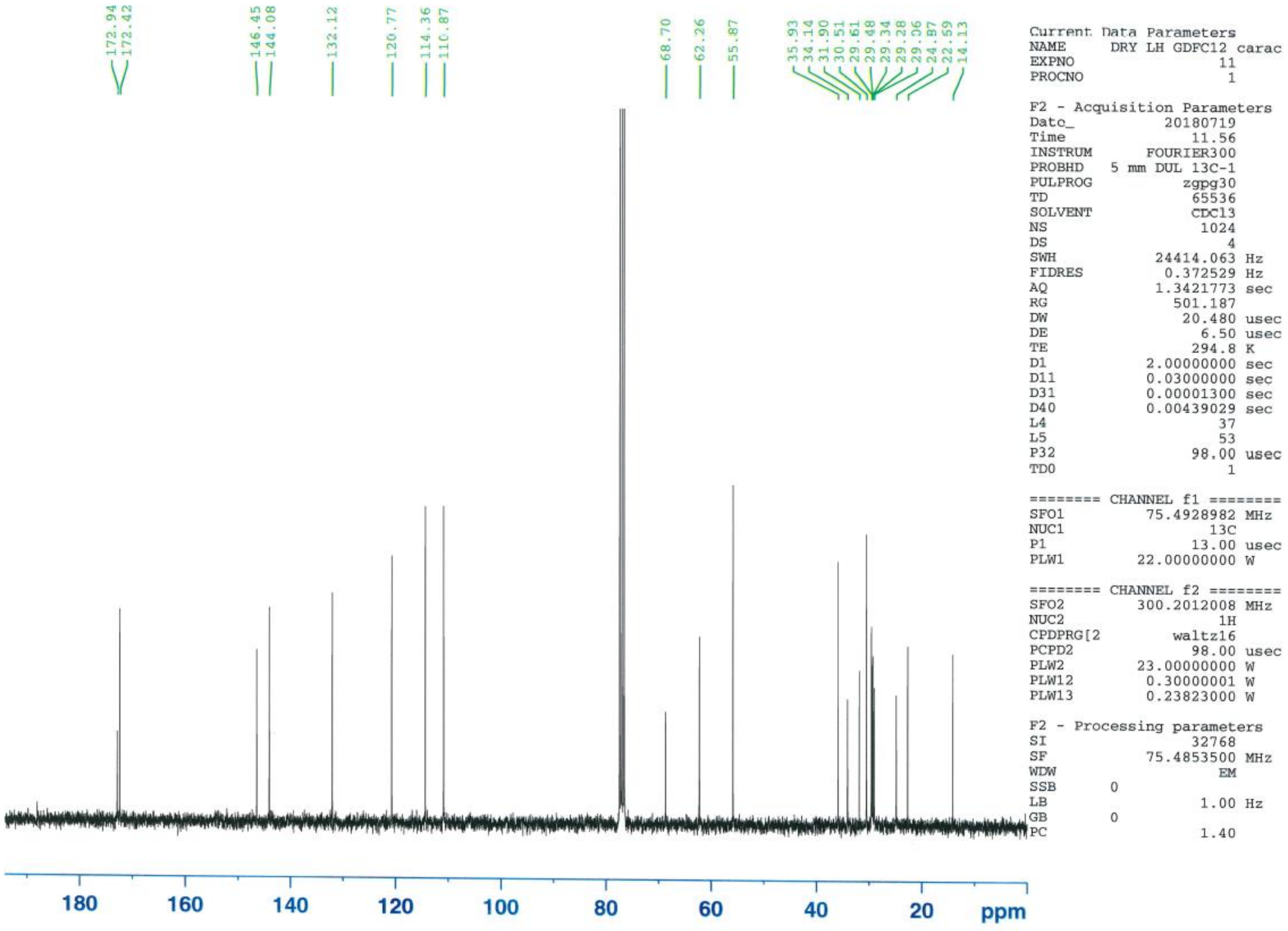

FT-IR spectra of GDF_{10}

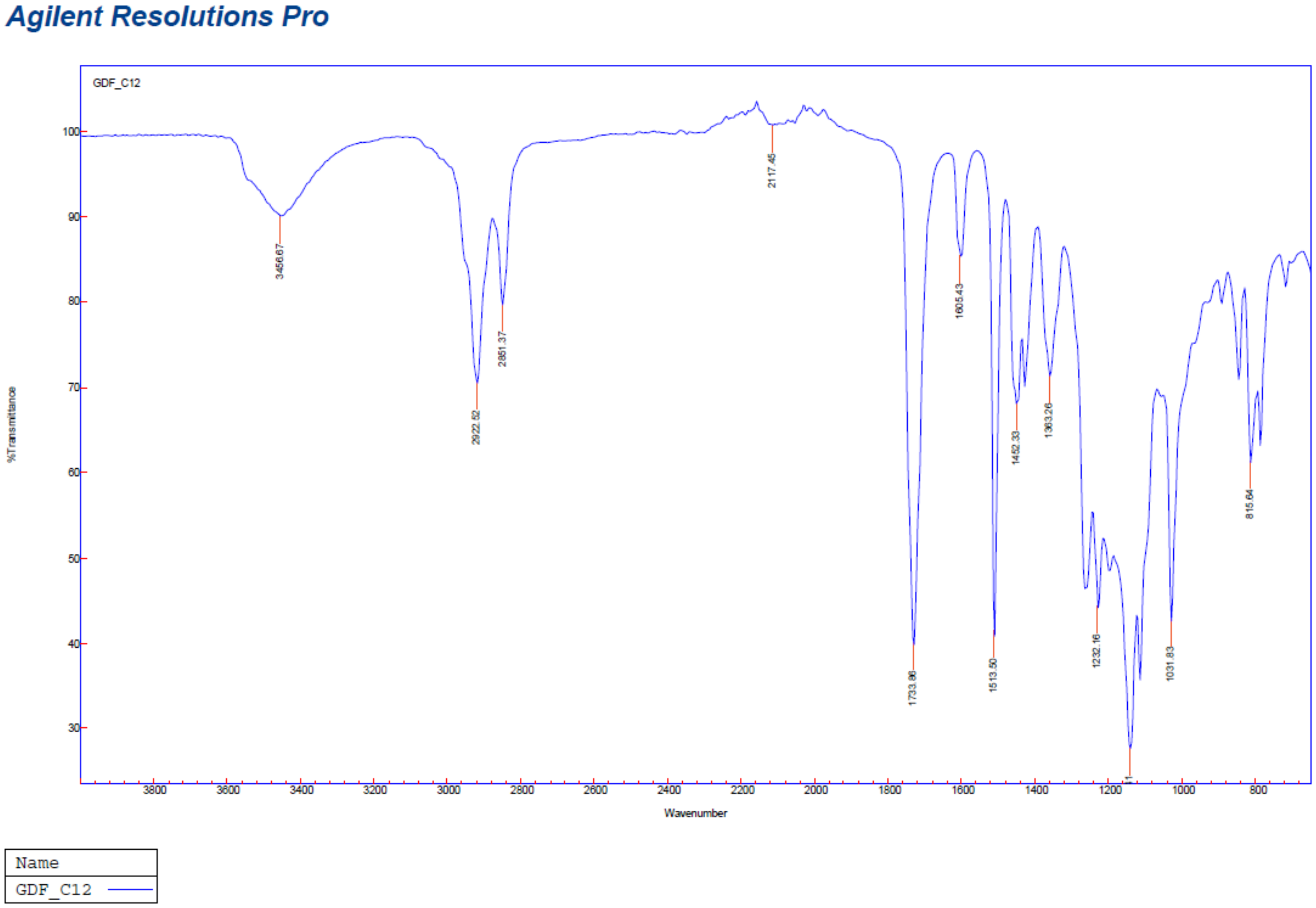

HRMS analysis of GDF_{10}

Elemental Composition Report

Single Mass Analysis
Tolerance = 5.0 PPM / DBE: min = -1.5, max = 50.0
Element prediction: Off
Number of isotope peaks used for i-FIT = 3

Monoisotopic Mass, Even Electron Ions
158 formula(e) evaluated with 1 results within limits (up to 50 closest results for each mass)
Elements Used:
C: 35-35 H: 0-200 O: 6-10 Na: 0-3 Al: 0-1 39K: 0-1 90Zr: 0-1

GDF_C12
17HR21 43 (1.409) AM (Cen,4, 80.00, Ar,5000.0,622.57,0.70,LS 20); Sm (SG, 1x1.00); Sb (5,40.00); Cm (41:43) 1: TOF MS ES+ 6.08e+003

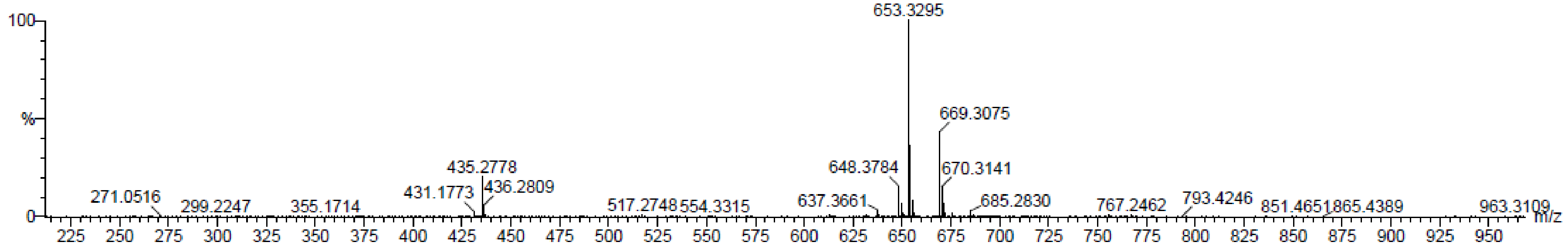

Minimum:				-1.5		
Maximum:		5.0	5.0	50.0		
Mass	Calc. Mass	mDa	PPM	DBE	i-FIT	Formula
653.3295	653.3302	-0.7	-1.1	10.5	19.4	C35 H50 O10 Na

TGA analysis of GDF_{10}

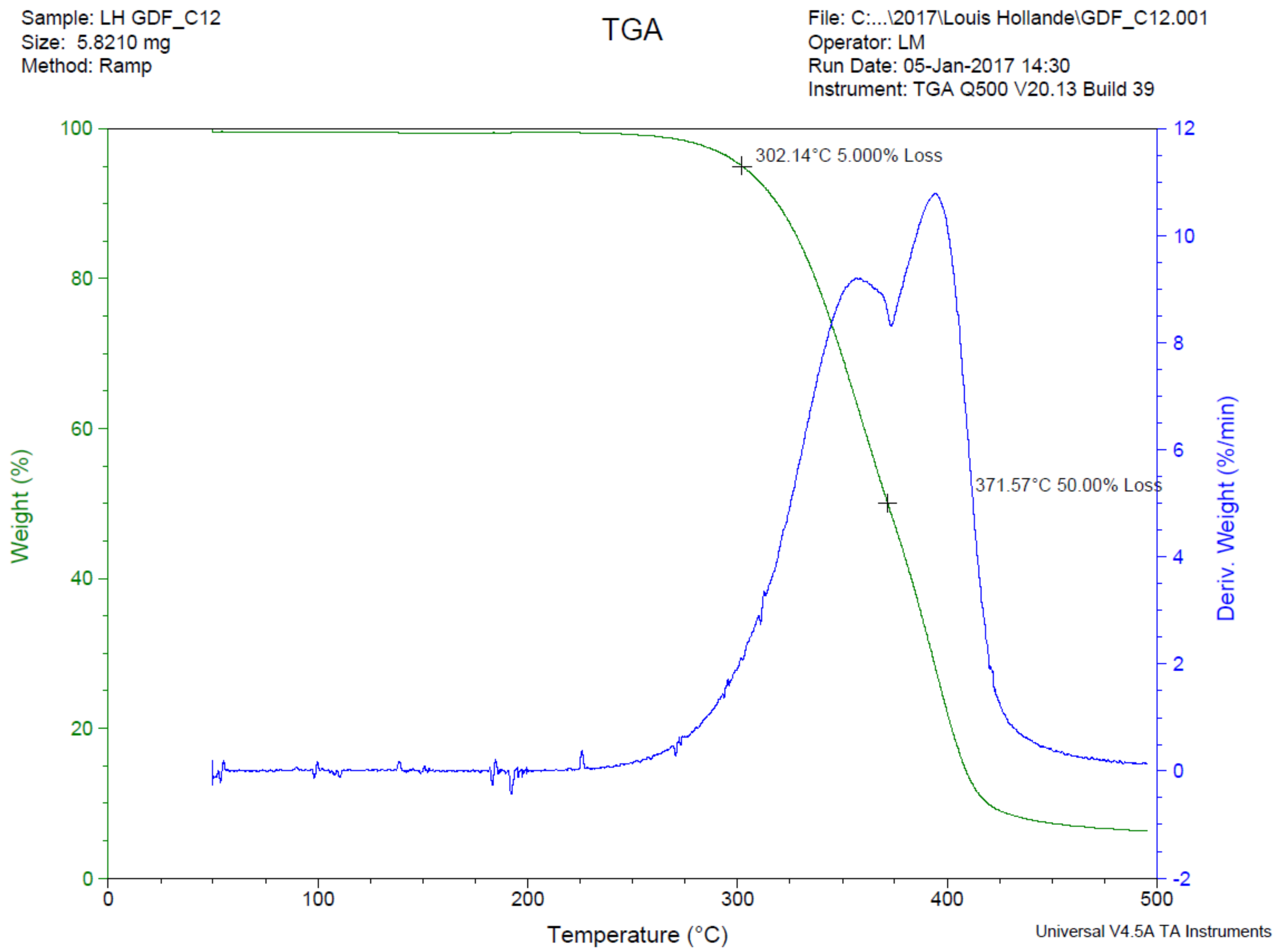

DPPH analysis (EC_{50}) of GDF_{10}

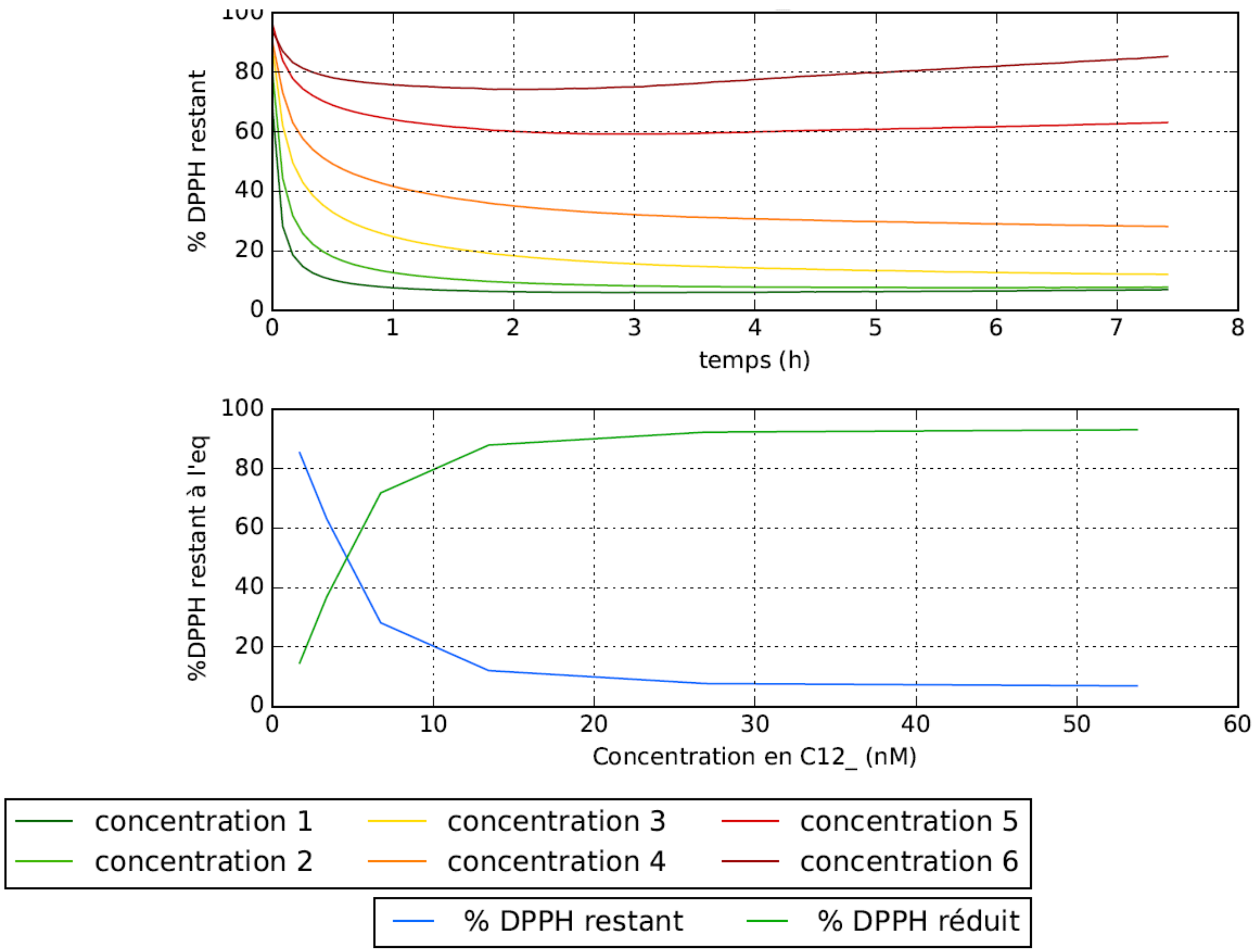

^{1}H NMR spectrum of GDF$_{14}$ (CDCl$_3$)

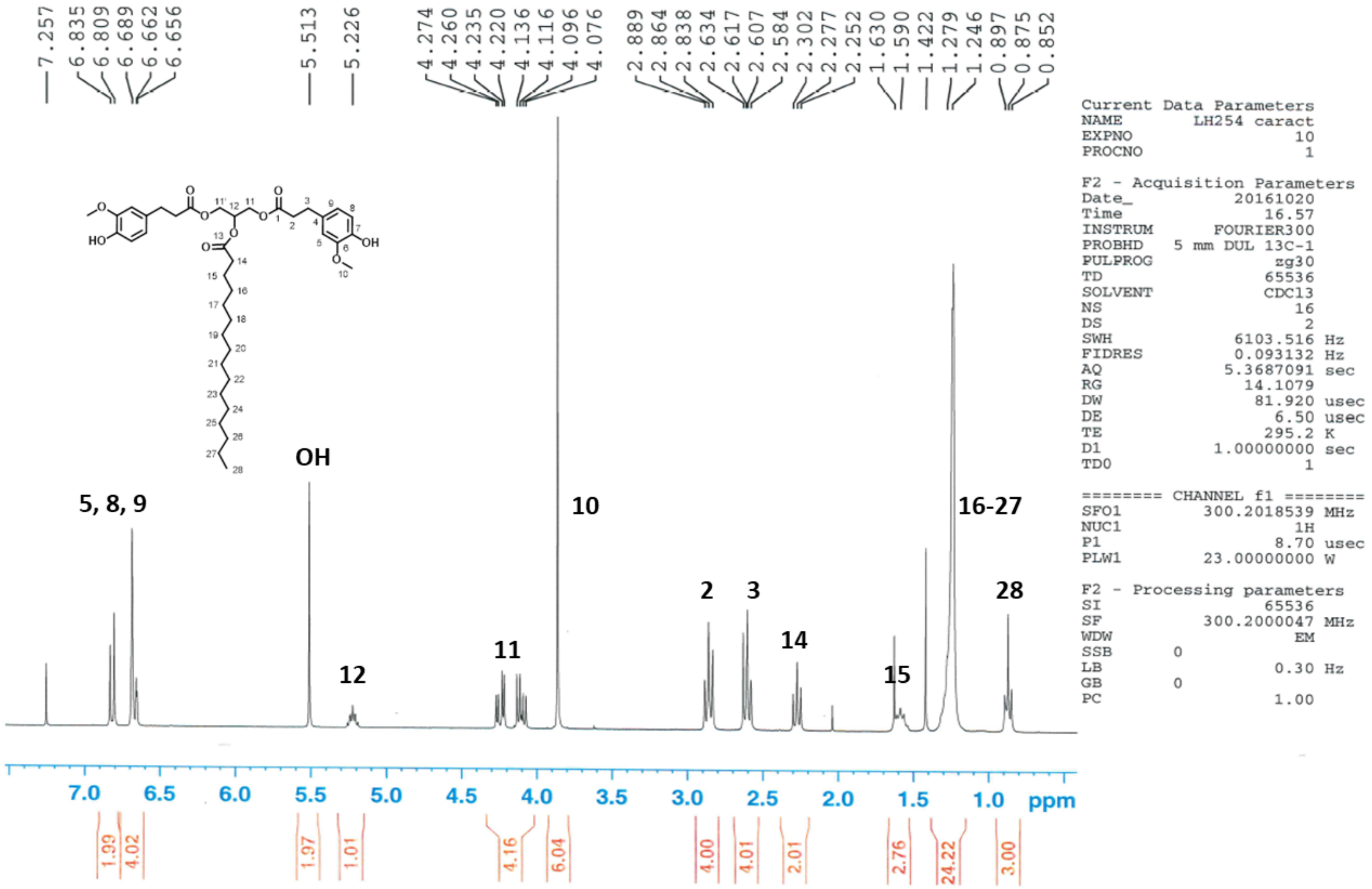

^{13}C NMR spectrum of GDF$_{14}$ (CDCl$_3$)

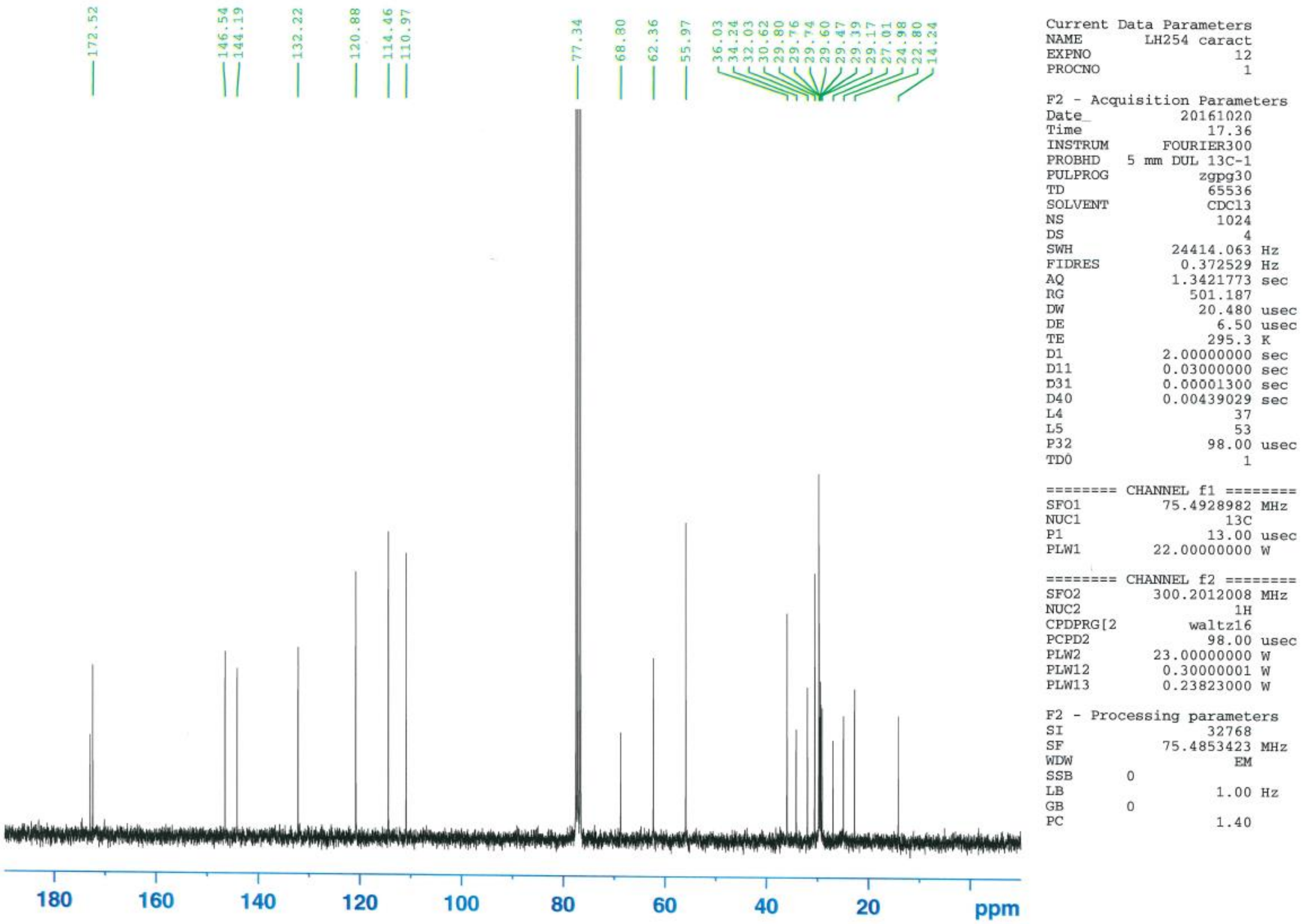

FT-IR spectra of GDF_{14}

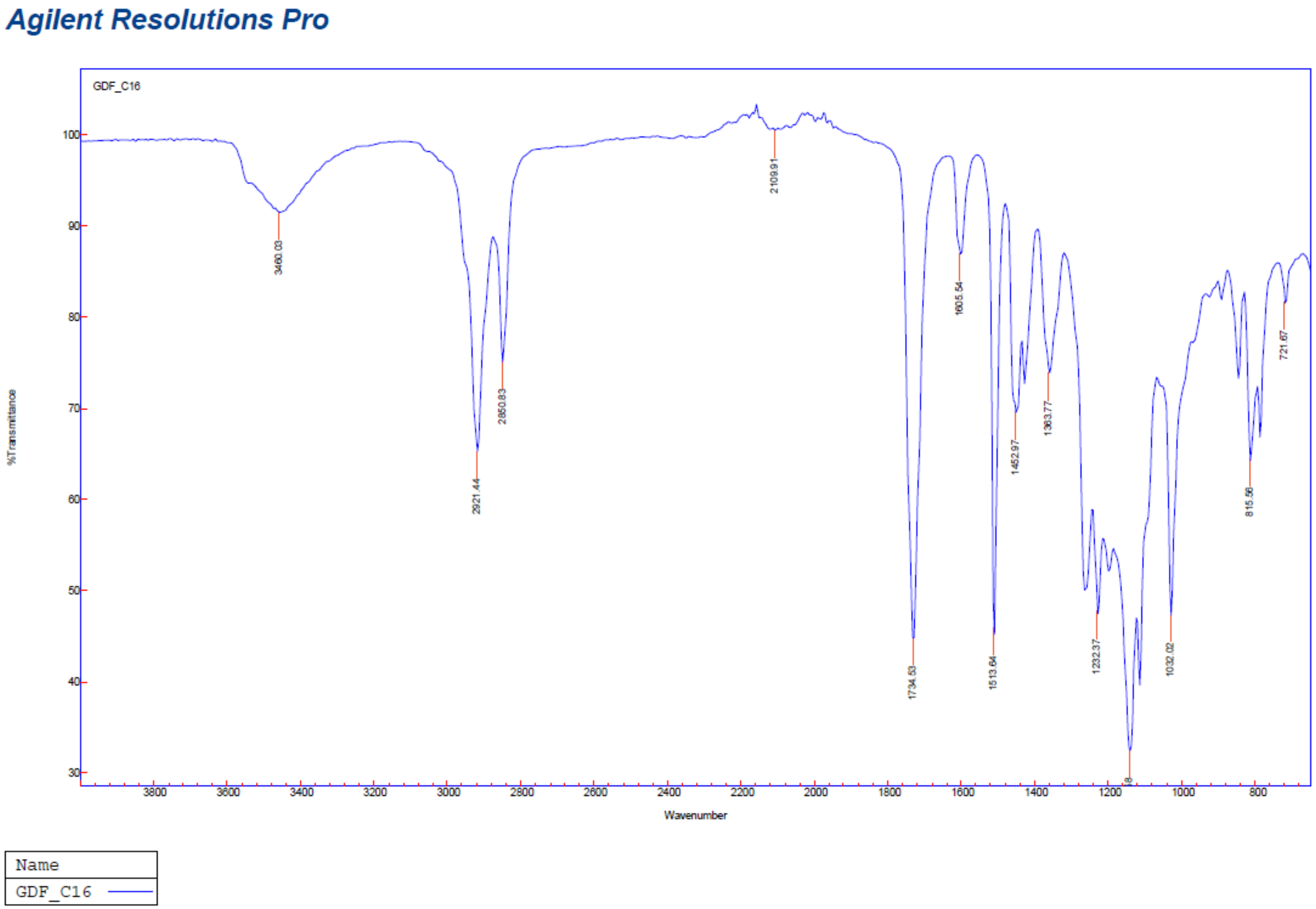

HRMS analysis of GDF_{14}

Elemental Composition Report

Single Mass Analysis

Tolerance = 5.0 PPM / DBE: min = -1.5, max = 50.0
Element prediction: Off
Number of isotope peaks used for i-FIT = 3

Monoisotopic Mass, Even Electron Ions
157 formula(e) evaluated with 1 results within limits (up to 50 closest results for each mass)
Elements Used:
C: 39-39 H: 0-200 O: 6-10 Na: 0-3 Al: 0-1 39K: 0-1 90Zr: 0-1

GDF_C16
17HR22 91 (2.965) AM (Cen,4, 80.00, Ar,5000.0,622.57,0.70,LS 20); Sm (SG, 1x1.00); Sb (5,40.00); Cm (91:94)

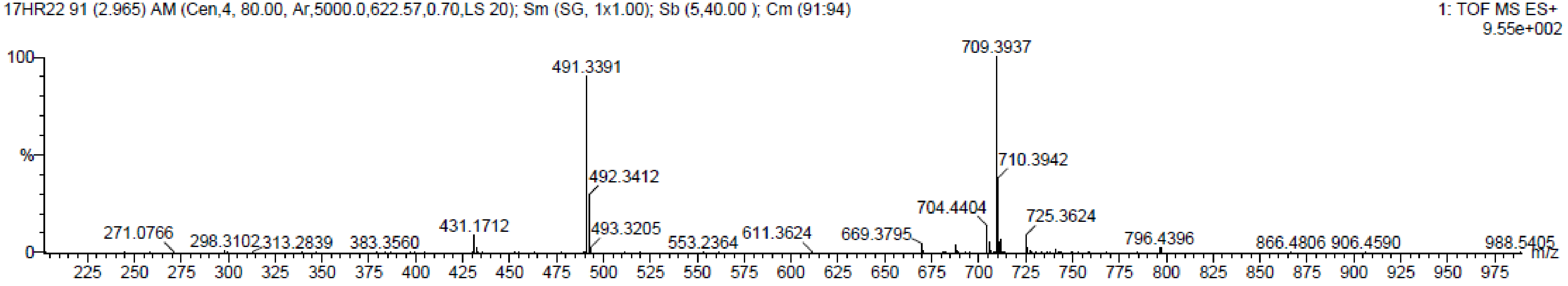

Minimum:				-1.5		
Maximum:		5.0	5.0	50.0		
Mass	Calc. Mass	mDa	PPM	DBE	i-FIT	Formula
709.3937	709.3928	0.9	1.3	10.5	27.7	C39 H58 O10 Na

TGA analysis of GDF_{14}

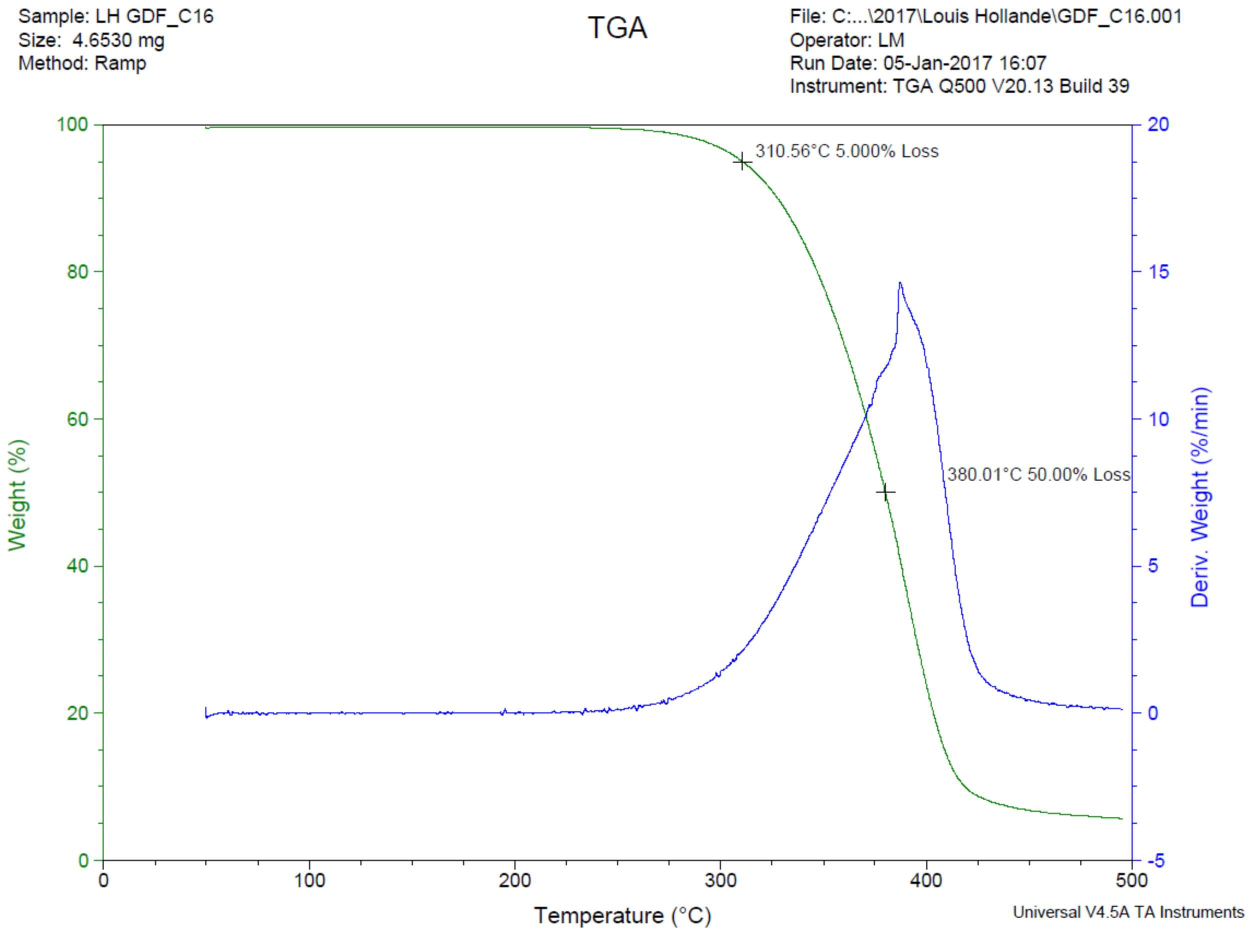

DPPH analysis (EC_{50}) of GDF_{14}

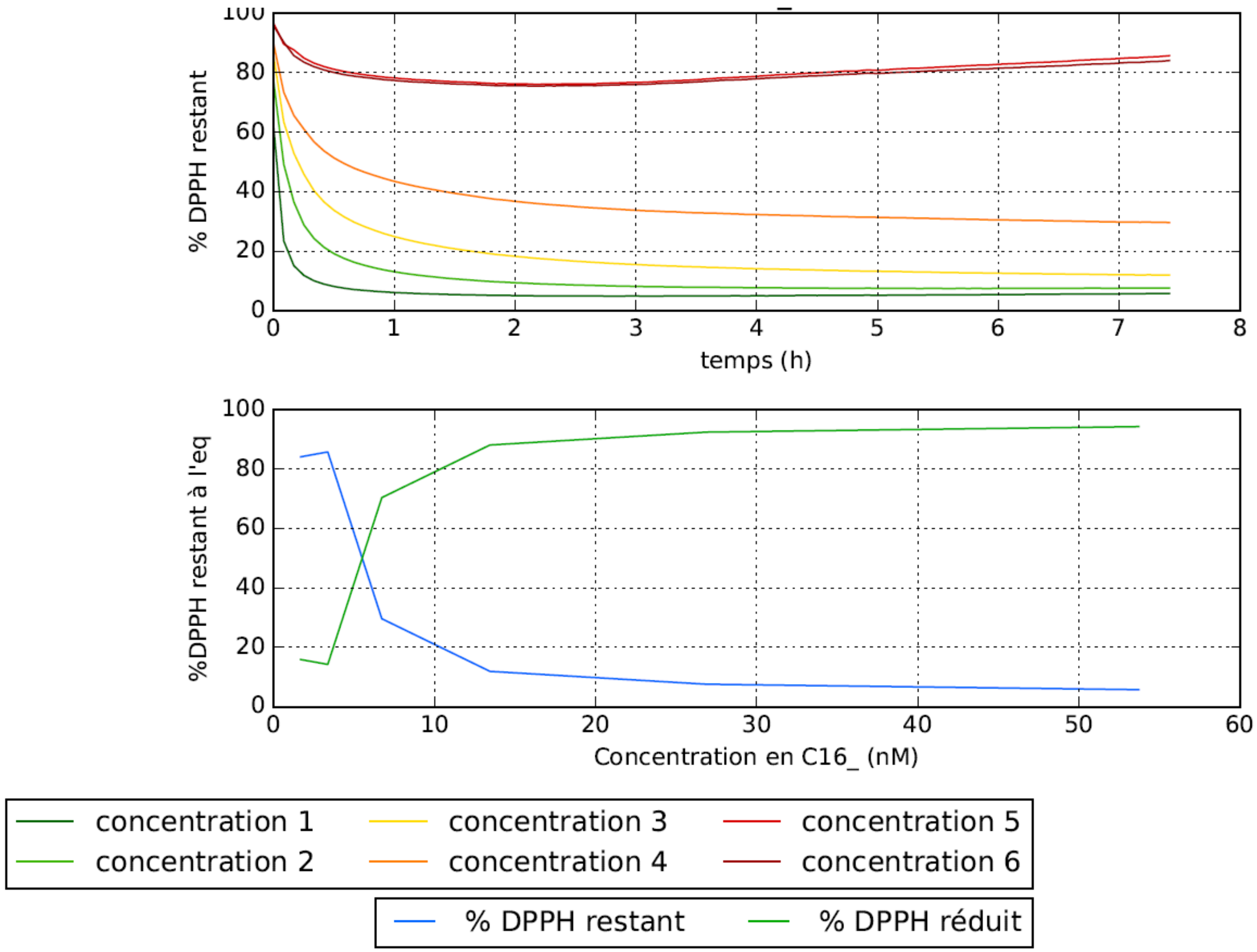

^{1}H NMR spectrum of GDF_{16} ($CDCl_3$)

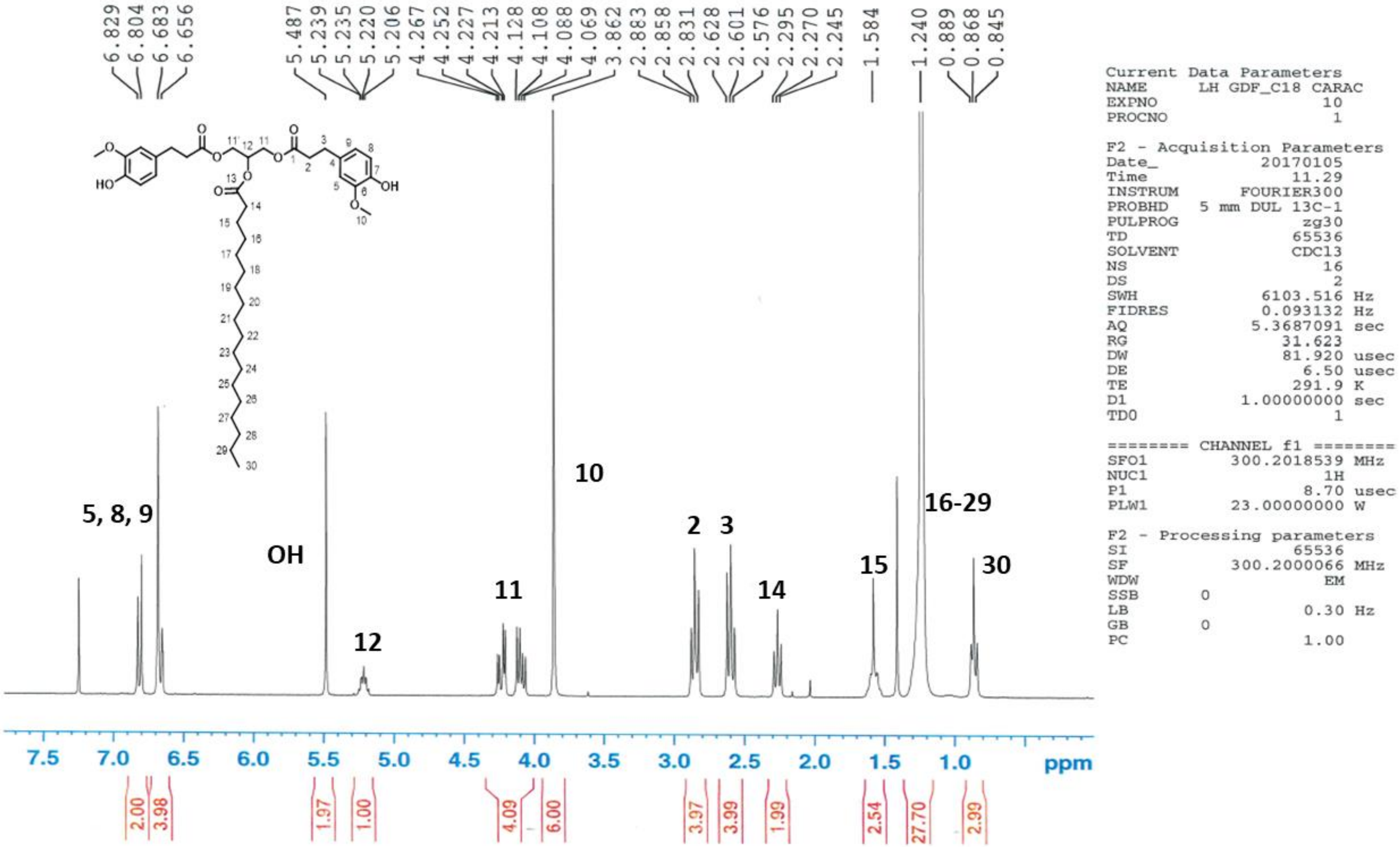

^{13}C NMR spectrum of GDF$_{16}$ ($CDCl_3$)

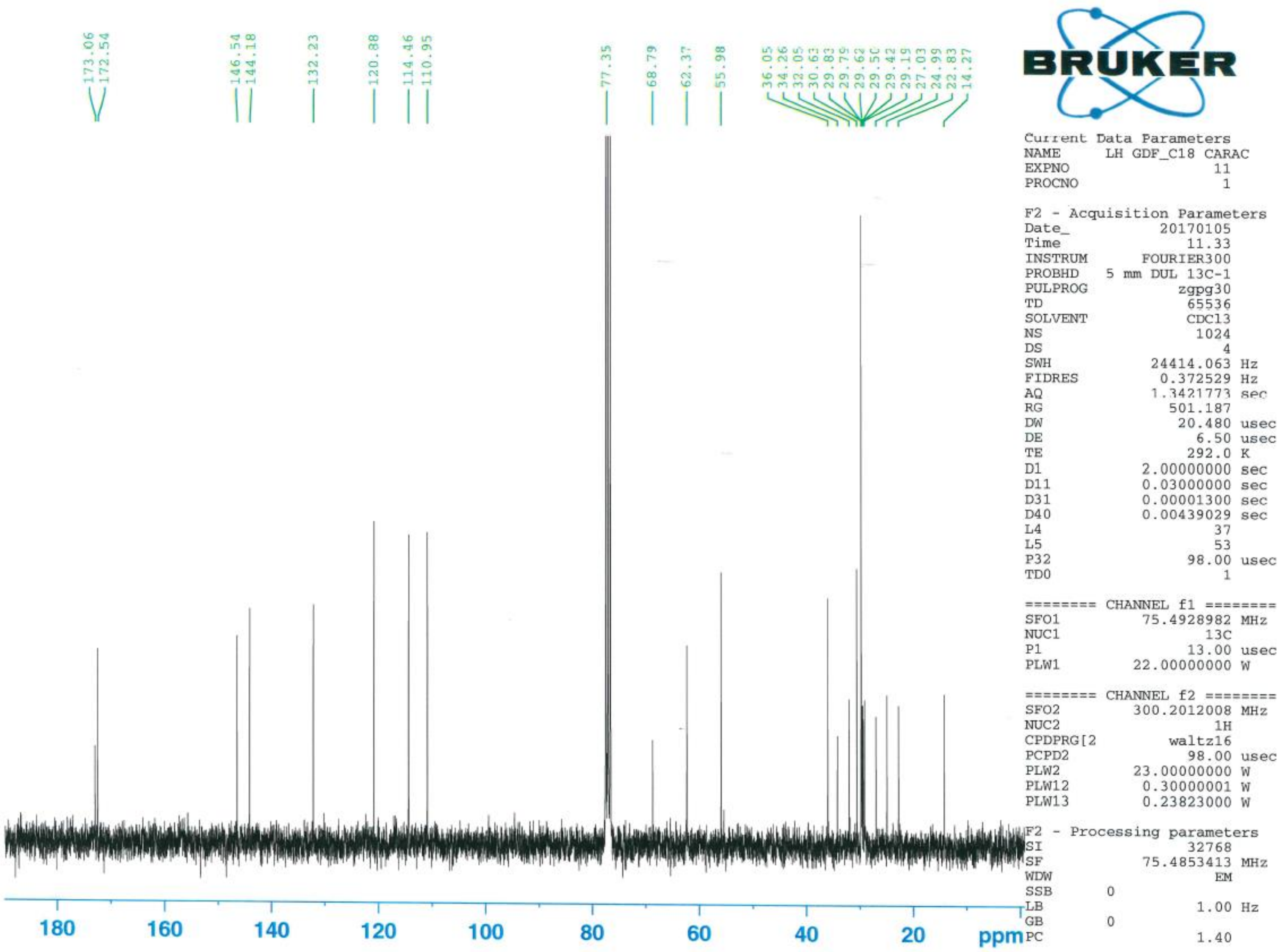

FT-IR spectra of GDF_{16}

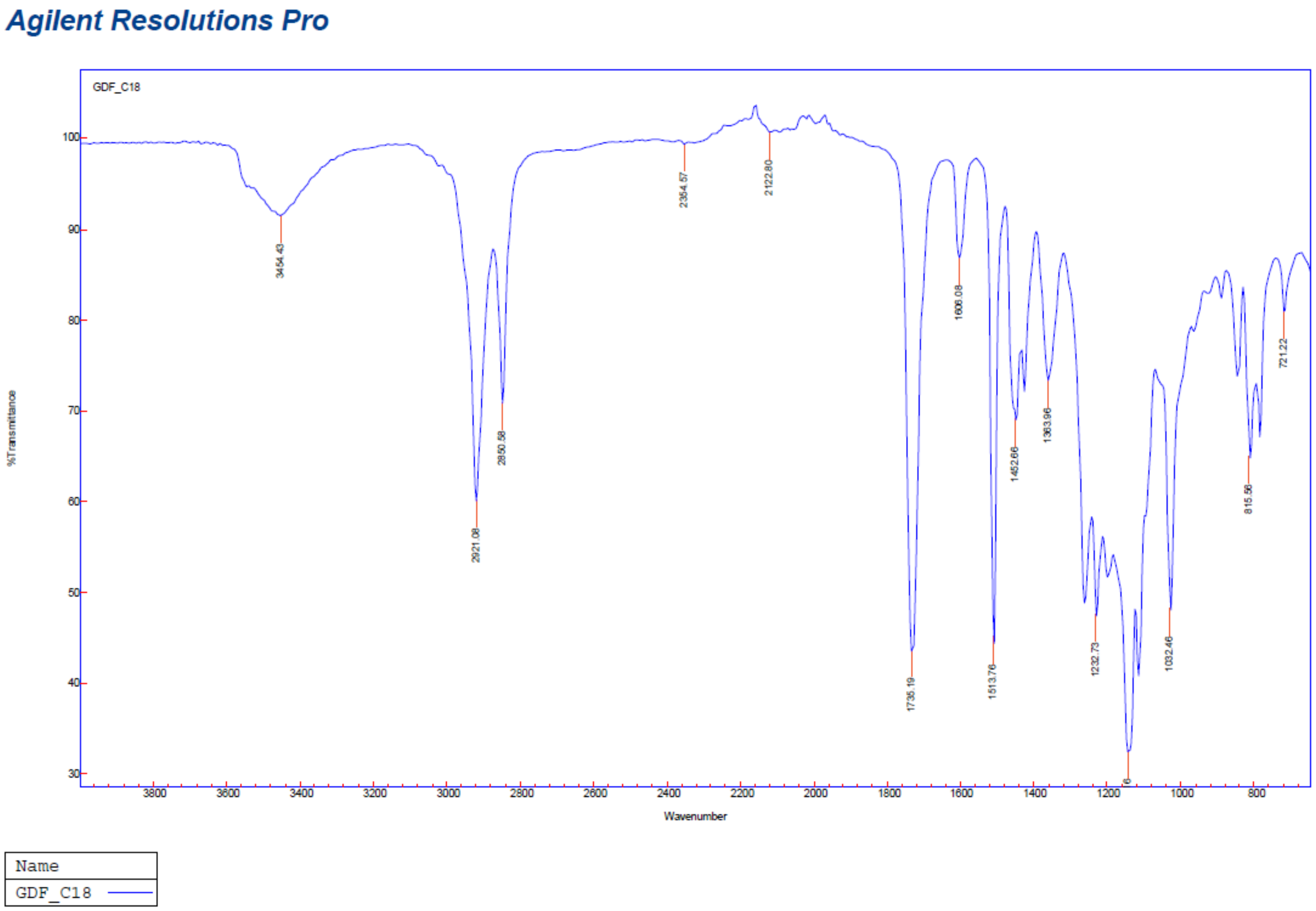

HRMS analysis of GDF_{16}

Elemental Composition Report

Single Mass Analysis

Tolerance = 5.0 PPM / DBE: min = -1.5, max = 50.0
Element prediction: Off
Number of isotope peaks used for i-FIT = 3

Monoisotopic Mass, Even Electron Ions
156 formula(e) evaluated with 1 results within limits (up to 50 closest results for each mass)
Elements Used:
C: 41-41 H: 0-200 O: 6-10 Na: 0-3 Al: 0-1 39K: 0-1 90Zr: 0-1

GDF_C18
17HR23 26 (0.839) AM (Cen,4, 80.00, Ar,5000.0,622.57,0.70,LS 20); Sm (SG, 1x1.00); Sb (5,40.00); Cm (23:26)

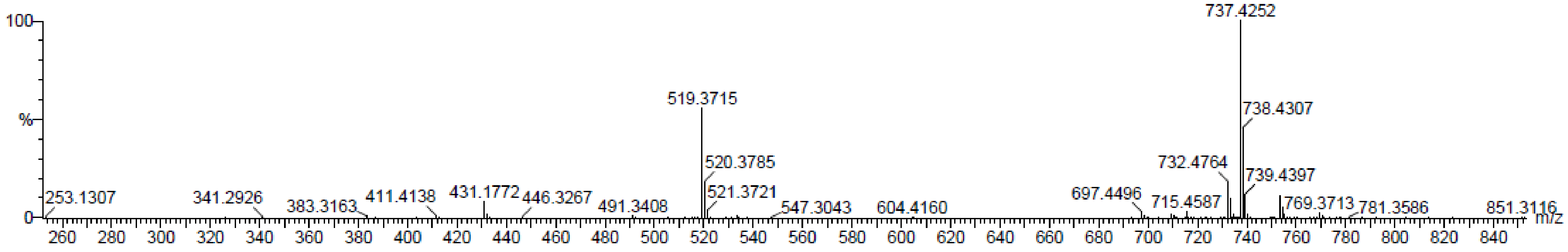

Minimum:				-1.5		
Maximum:		5.0	5.0	50.0		
Mass	Calc. Mass	mDa	PPM	DBE	i-FIT	Formula
737.4252	737.4241	1.1	1.5	10.5	0.9	C41 H62 O10 Na

TGA analysis of GDF_{16}

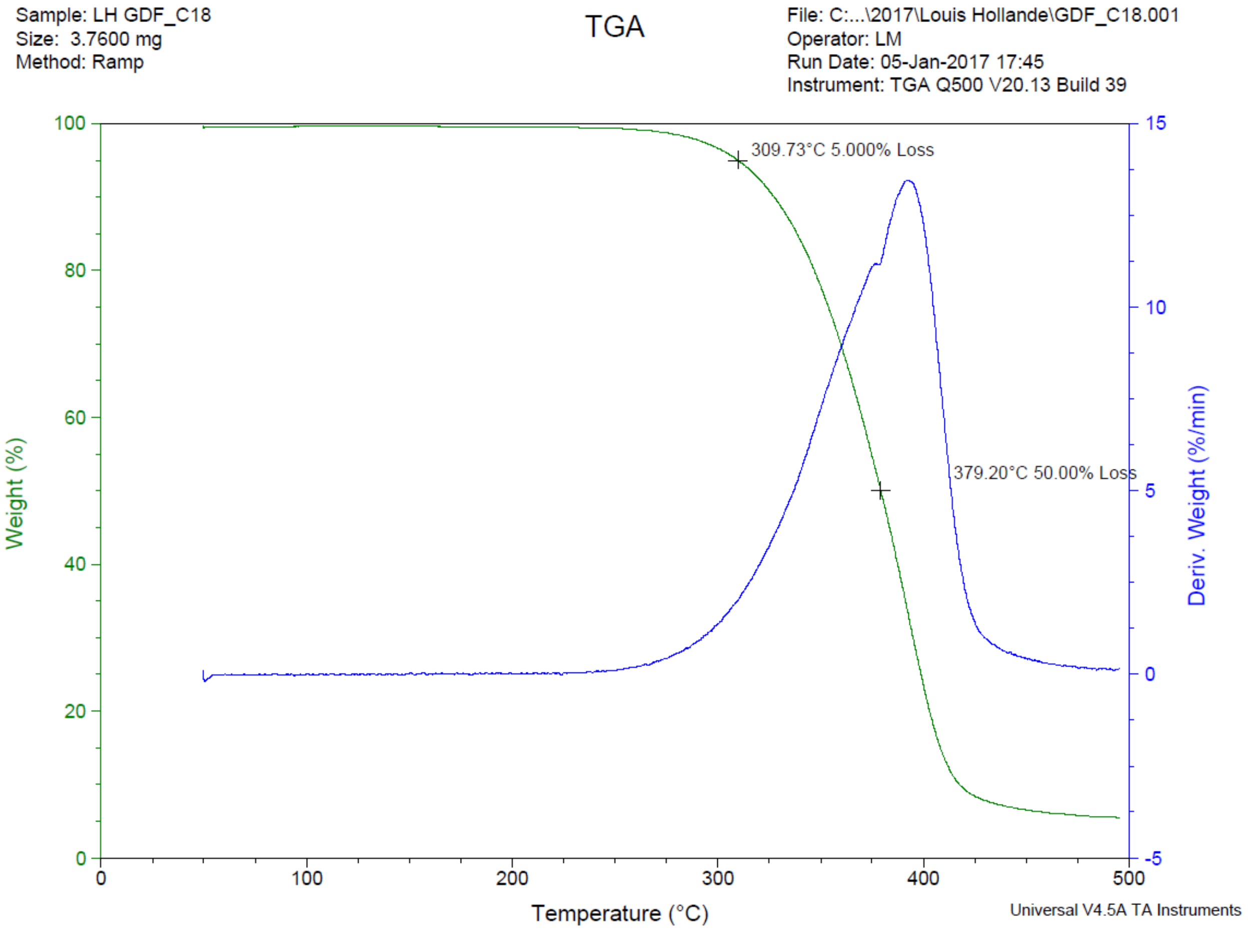

DPPH analysis (EC_{50}) of GDF_{16}

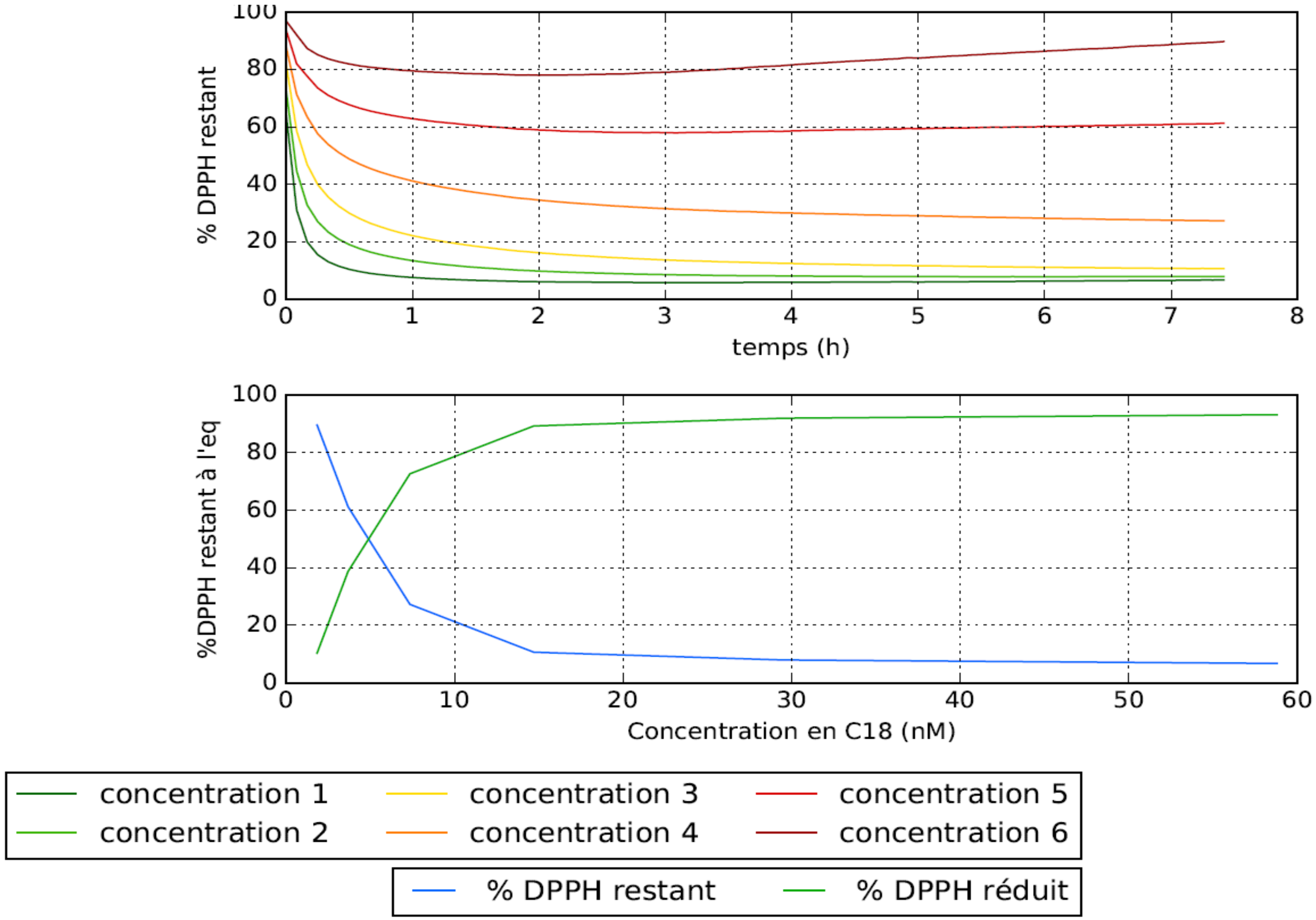

Kinetics behaviours at EC_{50} concentration for GDF_{10}, GDF_{14}, GDF_{16}, $Irganox_{1010}$, $Irganox_{1076}$

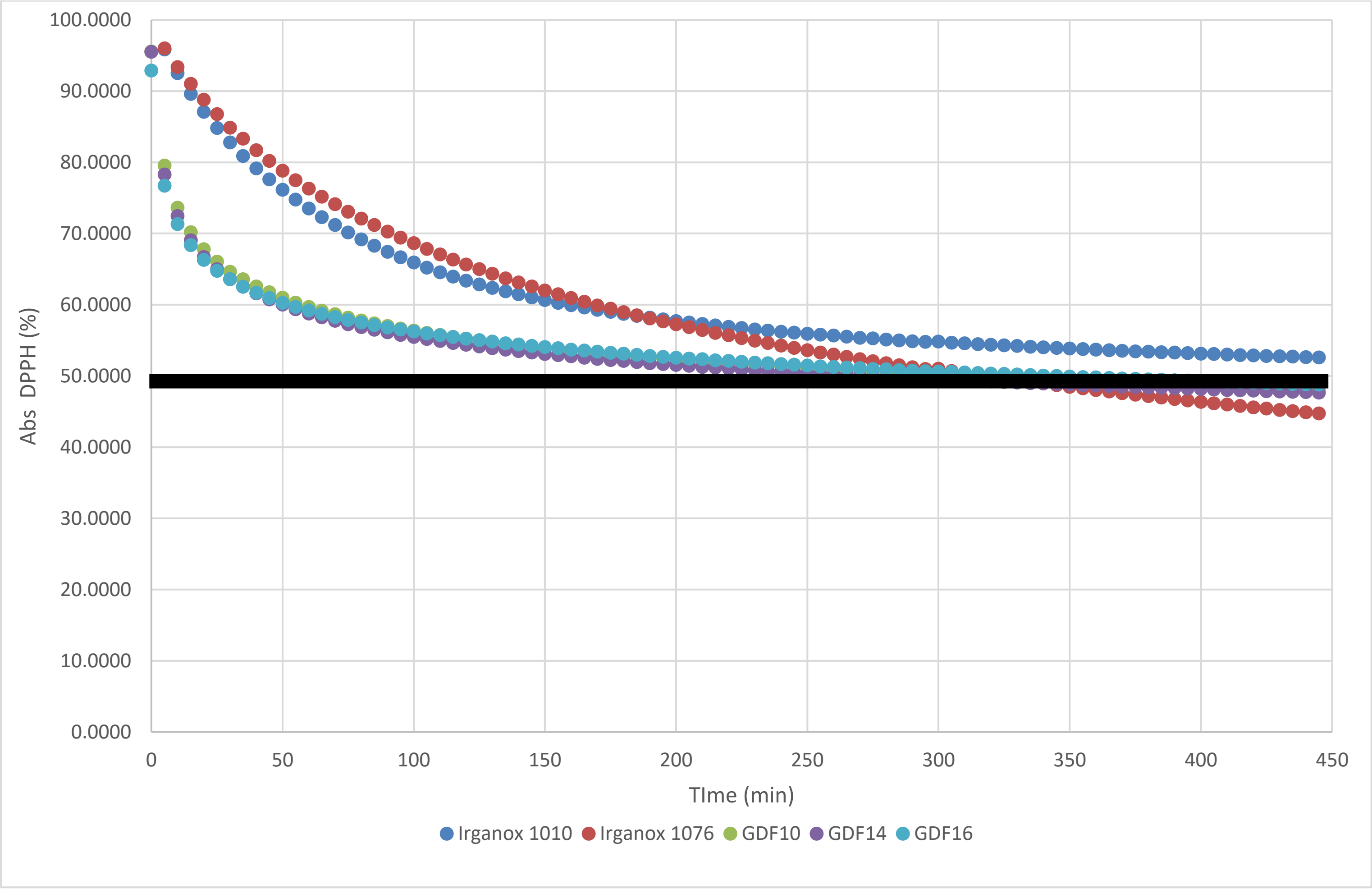

Supplement: Supplementary file 1 [file ijms-19-03358-s001.pdf]
